# Evaluation of methods for generative modeling of cell and nuclear shape

**DOI:** 10.1093/bioinformatics/bty983

**Published:** 2018-12-07

**Authors:** Xiongtao Ruan, Robert F Murphy

**Affiliations:** 1 Computational Biology Department, School of Computer Science; 2 Departments of Biological Sciences, Biomedical Engineering, and Machine Learning, Carnegie Mellon University, Pittsburgh, PA, USA

## Abstract

**Motivation:**

Cell shape provides both geometry for, and a reflection of, cell function. Numerous methods for describing and modeling cell shape have been described, but previous evaluation of these methods in terms of the accuracy of generative models has been limited.

**Results:**

Here we compare traditional methods and deep autoencoders to build generative models for cell shapes in terms of the accuracy with which shapes can be reconstructed from models. We evaluated the methods on different collections of 2D and 3D cell images, and found that none of the methods gave accurate reconstructions using low dimensional encodings. As expected, much higher accuracies were observed using high dimensional encodings, with outline-based methods significantly outperforming image-based autoencoders. The latter tended to encode all cells as having smooth shapes, even for high dimensions. For complex 3D cell shapes, we developed a significant improvement of a method based on the spherical harmonic transform that performs significantly better than other methods. We obtained similar results for the joint modeling of cell and nuclear shape. Finally, we evaluated the modeling of shape dynamics by interpolation in the shape space. We found that our modified method provided lower deformation energies along linear interpolation paths than other methods. This allows practical shape evolution in high dimensional shape spaces. We conclude that our improved spherical harmonic based methods are preferable for cell and nuclear shape modeling, providing better representations, higher computational efficiency and requiring fewer training images than deep learning methods.

**Availability and implementation:**

All software and data is available at http://murphylab.cbd.cmu.edu/software.

**Supplementary information:**

Supplementary data are available at *Bioinformatics* online.

## 1 Introduction

The shapes of cells vary during movement, development and in response to environmental changes such as drug treatment. Thus the study of cell shape is important to understanding fundamental biological processes. There are two different approaches for such studies: *discriminative* methods that try to capture just enough information about shapes to be able to distinguish previously defined classes, or *generative* methods that try to capture as much information as possible in order to be able to estimate the shape distribution of a population. For either approach, the native representation of cell shapes can be through a mask indicating which pixels in an image are contained within a cell, or through identification of points on the cell boundary; both of these are high dimensional and are therefore difficult to use directly for discrimination or generation. The discriminative task is typically accomplished by characterizing shapes using many different types of descriptive features: simple ones such as eccentricity or convex deficiency, or more complex features such as scale invariance feature transform (SIFT) (Lowe, 2004) and speed up robust feature (SURF) (Bay *et al.*, 2008). These may work well for a particular classification task (where shapes are quite different), but they are typically not useful for the generative task since trying to reconstruct a shape from such features can produce too many shapes that are not similar to the original in ways not captured by those features. For generative modeling, a higher dimensional, more complete description is typically used and the high dimensional cell shape representations are embedded to a low dimensional shape space. In this case, it is easy to visualize different cell shape populations and compare cell shapes of different cell types or states. The shape spaces can be viewed as a generative model of the cell shapes, from which every point in the space represents a possible cell shape, and a trajectory in shape space can represent shape dynamics (Johnson *et al.*, 2015b). There are two traditional approaches to building shape spaces. The first is a linear shape model based upon the parameterization of the cell boundary followed by linear methods, like PCA or ICA (Pincus and Theriot, 2007). The second uses manifold learning methods combined with deformation based methods. Large deformation diffeomorphic metric mapping (Beg *et al.*, 2005), is an example of the latter approach that has been successfully applied to nuclear and cell shape (Johnson *et al.*, 2015b; Rohde *et al.*, 2008; Roybal *et al.*, 2016). A significant disadvantage of diffeomorphic approaches is that the training process typically takes much longer than other methods.

A classical approach to performing shape analysis is using Principal Component analysis (PCA) on some kind of parameterization that captures shape variances (Dryden and Mardia, 2016). PCA is simple and fast, but it yields a linear model, and may not perform well for some tasks. Shape component analysis (SCA) was proposed by Lee *et al.* (2016), and seeks non-linear dimension reduction of 2D and 3D biological shape representations which were shown to be suitable for clustering.

**Fig. 1.**
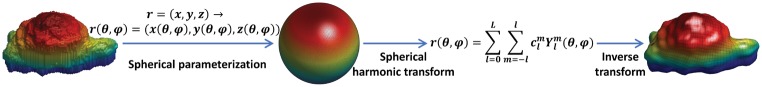
Illustration of Shape modeling using Spherical Harmonic transform. In the first step, the 3D surface mesh is mapped to a unit sphere. This results in spherical coordinates for each vertex in the original mesh. The spherical harmonic transform is then performed to get coefficients to represent the surface. A reconstruction of the original 3D surface mesh can be obtained by inverse transform followed by reversing the mapping

Though similar in many aspects, 3D shapes are usually challenging to model because unlike 2D shapes that can be easily represented with ordered arrays of outline landmarks that are comparable to each other, it is not trivial to represent 3D surfaces with similar features, as the shape variance has more degrees of freedom and there is no established ordering of surface coordinates in 3D space. For 3D shapes, a traditional approach is to represent shapes as surfaces and convert surfaces to shape descriptors. Among various descriptors, spherical harmonic descriptors are widely used (Kazhdan *et al.*, 2003; Tangelder and Veltkamp, 2008). The basic idea is to map the surface to a unit sphere and perform a spherical harmonic transform; the coefficients can be used as features (Kazhdan *et al.*, 2003) to compare different shapes or reconstruct the original shapes. An example scheme is shown in Figure 1. An advantage of the descriptor is that it is invariant under translations and scalings. However, the descriptors rely on the quality of the spherical parameterization, which may be challenging for complex cell shapes. Various methods have been developed for spherical parameterization (Brechbühler *et al.*, 1995; Shen and Makedon, 2006). Some previous work has applied the spherical harmonic representations to cell shapes (Ducroz *et al.*, 2012; Du *et al.*, 2013) and other biological shapes (Lee *et al.*, 2016).

Recently, deep learning techniques have shown powerful representation ability for various types of data, especially when a large number of samples are available. Unsupervised learning techniques such as autoencoders (Hinton and Salakhutdinov, 2006) and generative adversarial networks (Goodfellow *et al.*, 2014) provide powerful alternatives for building shape spaces that have recently been applied to build cell shape space or whole cell generative models (Johnson *et al.*, 2017; Osokin *et al.*, 2017).

As proposed by Rumelhart *et al.* (1986), an autoencoder learns some low dimensional representation such that the representation can be restored to the original input as accurately as possible. An autoencoder has an encoder and a decoder. The encoder uses the original data as input and learns some low dimensional representation. The decoder uses the low dimensional representation as input and produces an output with the same size as the original input. Training is performed to minimize the difference between the output and the original input. Many variants of autoencoders have been developed, such as convolutional autoencoder (Masci *et al.*, 2011), variational autoencoder (Kingma and Welling, 2013) and adversarial autoencoder (Makhzani *et al.*, 2015). An approach for representing cell and nuclear shapes based on adversarial autoencoders has been recently described (Johnson *et al.*, 2017).

An important consideration in shape modeling is whether size or orientation is considered implicitly or explicitly. Statistical shape analysis provides hierarchical definitions of shape configuration in terms of shape with or without size and/or rotation (Dryden and Mardia, 2016). However, deep learning methods usually use as input raw pixel images without removal of scale and orientations. To make fair comparisons between different methods, the total number of variables involved in constructing models (whether latent or explicit) must be considered.

In this paper we explore various methods for cell shape representation. Our focus is on accurate unsupervised representation, which is necessary for the creation of synthetic cell models, rather than supervised learning, since good discriminative models might not be adequate for generation.

## 2 Methods and datasets

### 2.1 Datasets and processing of data

**CYTO Challenge 2 dataset:** This dataset consists of raw 2D fluorescence microscope images from the CYTO 2017 Image Analysis Challenge (http://cytoconference.org/2017/Program/Image-Analysis-Challenge.aspx). The cell types are not specified in the dataset. We segmented the images from Challenge 2 using the watershed algorithm (Vincent and Soille, 1991). The images were downsampled by a factor of 2 and each cell placed in the center of a 512 × 512 image. We randomly chose 20 000 segmented images and used 10 000 images as the training set and the remaining images as the testing set. Examples from the dataset are shown in Supplementary Figure S1.

**HPA cell lines (HPA CL) dataset**: This dataset was downloaded from the Human Protein Atlas (HPA, https://www.proteinatlas.org/). It contains about 200 fluorescence microscope images for each of 10 cell lines. The images were processed in the same way as above. We randomly selected about 1500 segmented cells for each cell line to create a training set of 10 000 images and a testing set of 4900 images. Examples from the dataset are shown in Supplementary Figure S2.

**H1299 dataset**: This dataset was downloaded from the Kahn Dynamic Proteomics Database (Sigal *et al.*, 2006, 2007), which was also used in Johnson *et al.* (2015b) (also available from murphylab http://murphylab.web.cmu.edu/software/2015_MBoC_Cell_And_Nuclear_Shape/). It consists of 2D images from movies of H1299 non-small cell lung carcinoma cell lines expressing different proteins tagged with yellow fluorescent protein (YFP). We used the image processing pipeline from Johnson *et al.* (2015b), and obtained 6495 segmented cells, and we selected 5500 images as the training set and the remaining 995 images as the testing set. Examples from the dataset are shown in Supplementary Figure S3.

**Simulated Neuron-Like cell (SNL) 2D dataset**: To illustrate the modeling of different cell types other than typical squamous cells, we simulated neuron-like cells with long thin neurites; the simulation process is described in Supplementary Methods. For the dataset, the number of neurites was fixed as 2. We simulated 20 000 cells and randomly selected 10 000 cells as the training set and the other half as the testing set. Examples from the dataset are shown in Supplementary Figure S4.

**MCF7 dataset**: This was image set BBBC021v1 (Caie *et al.*, 2010), available from the Broad Bioimage Benchmark Collection (http://data.broadinstitute.org/bbbc/BBBC021/). We used the image processing pipeline from Johnson *et al.* (2015b), and picked 13 compounds as representative drugs for different mechanisms of action and combined their images together. This produced a dataset of 16 000 cells and we randomly selected 10 000 images as the training set and the remaining 6000 images as the testing set. Examples from the dataset are shown in Supplementary Figure S5.

**HeLa dataset**: This dataset (Velliste and Murphy, 2002) was downloaded from the Murphy Lab (http://murphylab.web.cmu.edu/data/3Dhela_images.html). It contains 500 3D fluorescence microscope images of HeLa cells with both cell and nuclear markers, with size 256×256×24. We randomly selected about 400 segmented cells as the training set and used the remaining 100 images as the testing set.

**SNL 3D dataset**: This is a synthetic dataset, based on the simulation process used for SNL 2D cells to generate a central slice, which was used to generate slices above and below. The detailed simulation method is described in Supplementary Methods. The images were finally converted to size 256×256×24. For this dataset, the number of neurites was randomly sampled from 0 to 2. 1500 cells were simulated, with 1200 cells as the training set and the remaining cells as the testing set.

**SNL NR2 3D dataset**: This is also a synthetic dataset, with the same generation method as SNL 3D dataset, except the number of neurite was fixed as 2. 1000 cells were simulated with 800 as the training set and 200 as the testing set.

### 2.2 2D shape outline extraction

For 2D outline-based PCA (outline PCA) and SCA models, evenly-spaced outline coordinates were extracted from the binary cell and/or nuclear shape images as previously described (Pincus and Theriot, 2007). Starting from the leftmost point in the boundary, evenly-spaced points were traced along each outline. We used 2000 points for outlining cell shape and 1000 points for nuclear shape. The ordered outline was further processed by alignment and/or size normalization as described below.

### 2.3 Outline PCA

The method was basically the same as in Pincus and Theriot (2007). First, the cell or nuclear images were converted to a shape vector as described above. Outline coordinates were centralized and aligned along the major-axis. Then, PCA was applied to the vectors for all cells or nuclei. The mean ***μ***, and the principal vectors $U_{k}=[u_{1},u_{2},\ldots ,u_{k}]\in R^{nd\times k}$ were kept as the model parameters (where *k* was the desired number of latent dimensions). For a shape $x\in R^{d\times n}$, where *d* is the dimension of the shape, and *n* is the number of outline points, the low dimensional representation was $b=U_{k}^{T}{\left(vec(x)-\mu \right)}^{}$, where *vec(·)* is the operator to reshape a matrix to a column vector. The reconstructed shape was $\hat{x}=U_{k}b+\mu$, where $\hat{x}$ is then reshaped to *d *×* n*. The reconstruction errors are computed using x and $\hat{x}$ as described below.

### 2.4 SCA

The SCA model (Lee *et al.*, 2016) is a variant of outline PCA method. It requires converting the outline coordinates to a preshape, which is defined as a shape with scale and location removed. Briefly, the matrix $C=\sum_{j}x_{j}^{T}x_{j}$ was first calculated, where $x_{j}\in R^{d\times n}$ is the *j*-th preshape. Then, eigen decomposition was performed over C and the first *r* eigenvectors were used and put as columns into a matrix R. The representation was the vector normalized by the Frobenius norm, ${\hat{x}}_{j}=x_{j}R/||x_{j}R|{|}_{F}$. In the original paper, the model was only used for reconstruction of training data. Here we make an extension of the method to the representation and reconstruction of testing data. Given a preshape $x_{o}$, the representation is $b_{o}=x_{j}R/||x_{j}R|{|}_{F}$ and the reconstruction is ${\hat{x}}_{o}=b_{o}R^{T}$. ${\hat{x}}_{o}$ and $x_{o}$ were used for reconstruction error calculation.

### 2.5 Spherical harmonic descriptor based models

#### 2.5.1 Spherical harmonic transform

Similar to the Fourier transform, the spherical harmonic (SPHARM) transform provides a representation in an orthogonal space. In order to use it, the original shape, represented as a mesh of equal area elements converted from a voxel image, must first be mapped to a sphere. In theory, any genus-0 shape (one without holes or ring structure) can be so mapped. However, the quality of the mapping is critical, as will be discussed below. In the transform, the basis is the solution of the spherical harmonic function (Press *et al.*, 2007).

It has the form:

(1) $$Y_{l}^{m}(\theta ,\phi )=\sqrt{\frac{2l+1}{4\pi }\frac{(l-m)!}{(l+m)!}}P_{l}^{m}(\text{cos}\;\theta )e^{im\phi }$$

where $P_{l}^{m}$ are associated Legendre polynomials with form:

(2) $$P_{l}^{m}(w)=\frac{{\left(-1\right)}^{m}}{2^{l}l!}{\left(1-w^{2}\right)}^{\frac{m}{2}}\frac{d^{m+l}}{dw^{m+l}}{\left(w^{2}-1\right)}^{l}$$

The above equation is based on the spherical parameterization of the original surface, which means all points in the surface are mapped to the unit sphere as r(θ,ϕ)=(x(θ,ϕ),y(θ,ϕ),z(θ,ϕ)). After parameterization, each point can be (approximately) represented as

(3) $$r(\theta ,\phi )=\sum_{l=0}^{L}\sum_{m=-l}^{l}c_{l}^{m}Y_{l}^{m}(\theta ,\phi )$$

where *L* is the maximum order, and $c=(c_{0}^{0},c_{1}^{-1},\ldots ,c_{l}^{l})\in C^{L^{2}}$ is the coefficient vector, which is used as the shape descriptor (SPHARM descriptor). The descriptor can be used to retrieve the original shapes via reverse transform or used as features for comparison or classification of shapes. The accuracy of the descriptor is controlled by the maximum order of spherical harmonics *L*. The higher the maximum order, the more accurate the representation is, yet the higher the computing cost is. For the methods in the following section, the SPHARM features were reduced to a specified number of dimensions using PCA. For reconstruction, vertices in the sphere were chosen to be evenly distributed so that the vertices on the reconstructed shapes are in general evenly spaced.

#### 2.5.2 SPHARM descriptor based methods

##### 2.5.2.1 SPHARM-MAT

SPHARM-MAT is MATLAB software intended to analyze brain images (Shen *et al.*, 2009). The spherical parameterization method in the software is the Control of Area and Length Distortions (CALD) algorithm (Shen and Makedon, 2006). Though the software is for brain images, it has been used to analyze cell images (Ducroz *et al.*, 2012; Du *et al.*, 2013). Here, we use the software to perform spherical parameterization, and use the spherical harmonics descriptors as features for shape modeling methods.

##### 2.5.2.2 Weighted SPHARM

Weighted SPHARM (WSPHARM) was developed by Chung *et al.* (2007). It is a generalization of SPHARM where exponential weights for the basis are applied. Compared to the traditional SPHARM method, the representation converges faster and ringing artifices can be reduced significantly. For the spherical parameterization, we first smoothed the voxel image and converted the image to a quadrilateral mesh, and then used the initialization method in SPHARM-MAT to get a spherical mapping. After that, the descriptor was calculated with the weighted-SPHARM package. The reconstruction was also calculated with the package.

##### 2.5.2.3 SPHARM-PDM

SPHARM-PDM was developed by Styner *et al.* (2006) to perform statistical shape analysis of brain structures, based on the algorithm in Brechbühler *et al.* (1995). It was also used as parameterization method in a previous cell analysis pipeline (Du *et al.*, 2013). The software package contains 4 steps: preprocessing, spherical parameterization, spherical harmonic transform and alignment and statistical analysis. We found a small bug in the spherical parameterization function that may cause it to be stuck in an infinite loop, which we fixed by setting a stopping criterion.

##### 2.5.2.4 SPHARM-RPDM

As discussed in the Section 3, we observed that the SPHARM-PDM package frequently failed in the spherical parameterization step, especially for complicated shapes such as cells with neurites. We therefore produced a Matlab package based upon SPHARM-PDM, which we refer to as SPHARM-RPDM (for robust SPHARM-PDM), that has a number of modifications as summarized below. First, we perform very rigorous topology fixation via image processing. This consists of checking the character numbers after converting the voxel images to surfaces, and if the character number does not fulfill that of the genus-0 surface, performing image closing operations with increasing kernel size to remove holes, small gaps or small protrusions (until the requirement is met). Second, for the initial mapping, instead of picking two points with the largest and smallest Z-coordinates as two poles, we treat the mesh as a graph and find the two points that form the diameter of the graph (the largest shortest distance in the graph). This guarantees that the two poles will be as farther away from each other as possible, which can reduce the distortion of initial parameterization. We also adapt the method in SPHARM-MAT to get the initial parameterization. Third, instead of using the finite difference method to calculate the Jacobian matrix and gradient in the algorithm, the analytic ones using symbolic computing are used (which is much more efficient and accurate). Fourth, we use better linear solvers from Matlab for the update, clip the gradient and Jacobian to make sure the update does not diverge, and use constrained update methods to make sure the system is stable. We implemented a simple and efficient algorithm for constrained least squares with the ADMM (alternating direction method of multipliers) framework, which works if the condition number of the Jacobian is large. Fifth, we implemented a method to check whether the spherical parameterization is successful by checking the error between the reconstructed and original shapes. If it fails, we smooth the surface and use a different initialization method, i.e. the original ones in SPHARM-PDM or SPHARM-MAT, and redo the parameterization.

#### 2.5.3 Shape alignment with SPHARM descriptors

To make different parameterizations comparable and to remove rotation in shapes (especially good for small-scale datasets), the shapes can be aligned. As described previously (Shen *et al.*, 2009; Styner *et al.*, 2006), there are two main approaches for alignment: alignment based on major axis or first-order ellipse (FOE). We used FOE here. Cells were aligned (rotated) only in the XY-plane since that is the plane of the substrate to which they are attached. To do this, we adapted the FOE alignment implementation in SPHARM-MAT. However, we rotated only around the Z-axis so that the major axis of the ellipse was in the XZ-plane. The skewness of the projection in the XY-plane was calculated, and if the X-axis skewness was negative, the cell was rotated $180^{{}^{\circ}}$ around the Z-axis, so that all cells were aligned in the same direction. In color figures showing example shapes (available online), each face in a surface is colored by its order in the z-axis.

### 2.6 Diffeomorphic model

We used the diffeomorphic modeling approach described previously (Rohde *et al.*, 2008) and used CellOrganizer (http://cellorganizer.org/) functions to build the model. Cells were aligned using major axis rotation. The model for the training set was inferred by matrix completion from the distances between 250 randomly chosen shapes as reference points. For prediction, the coordinates for test shapes were inferred from their diffeomorphic distances to reference points, and the inferred coordinates were used to produce reconstructed shapes.

### 2.7 Deep autoencoders

#### 2.7.1 Basic frameworks

General descriptions of the different autoencoders examined are presented below; the details of network structures and settings are described in Supplementary Methods. The first three kinds of autoencoders used images as input, while the last used a feature representation.

**Valina autoencoder (AE):** This autoencoder uses residual network blocks (He *et al.*, 2016) as its basic blocks, where there is a shortcut connection between intermediate and final layers in a block. The encoder and decoder consist of multiple residual network blocks and they are basically symmetric. The latent representation is the encoded output of encoders with specific dimensions (latent dimensions that control the capacity of model). Because the image size varies across datasets, the network structures were slightly different.

**Variational autoencoder (VAE):** The setting for Variational autoencoders follows that in Kingma and Welling (2013). The basic structure was similar to the structure of Valina autoencoders, except that the outputs of residual network blocks in the encoder were used as input for the two different fully connected layers to get mean and log-variances as in variational autoencoder.

**Structure reference autoencoder (SRAE):** This autoencoder was proposed in Johnson *et al.* (2017). It aims to learn the framework (cell/nuclear shape) of a cell, and is variant of adversarial autoencoder (Makhzani *et al.*, 2015). We followed the structure and parameters in their paper, with some adaption to our datasets because of different image sizes.

**Outline autoencoder (O-AE):** This autoencoder consists of stacks of fully-connected layer, batch normalization and ReLu layers, with the sum of squared difference between the original and reconstructed shapes used as the loss function. The input was either outlines for 2D shapes or spherical harmonic descriptors for 3D shapes.

#### 2.7.2 Loss function and image augmentation

Hinge loss was used as the loss function for valina autoencoders. For variational autoencoders, hinge loss was used as the reconstruction loss function and Kullback-Leibler divergence was used for matching the distribution, as originally described (Kingma and Welling, 2013). For the structure reference autoencoder, the loss functions and other parameters followed those in Johnson *et al.* (2017).

Image augmentation was used to preprocess input images for the autoencoders in order to improve the training process. The image augmentations included random translation and random rotation. For 3D shapes, random rotation was only allowed in the XY-plane.

### 2.8 Reconstruction error

We used Hausdorff distance throughout our studies for measuring the quality of shape reconstruction. It has the following form:

(4) $$D(X,Y)=\max(\underset{x\in X}{\max}\underset{y\in Y}{\min}d(x,y),\underset{y\in Y}{\max}\underset{x\in X}{\min}d(x,y))$$

where *X* and *Y* are two sets, and *d*(*x*, *y*) is the distance metric (Euclidean in our case). Intuitively, Hausdorff distance finds the worst match between a pair of points for two surfaces, making it a more strict criterion for matching two curves or surfaces than root mean square error, which has been used previously (Du *et al.*, 2013; Lee *et al.*, 2016). For 2D images, we converted the original and reconstructed images (if the reconstructed shape is an image) to outline landmarks, and calculated Hausdorff distances. For 3D images, we converted volume images to meshes, and calculated Hausdorff distances between the original and reconstructed meshes. We used the mean Hausdorff distance of all shapes in a dataset as the reconstruction error for a given method.

For the performance of joint modeling methods (described in Section 2.10), the average reconstruction errors of the cell and nuclear shapes were calculated. The overall error was the average of the average cell and nuclear shape errors (it thus equally weighted errors in each).

### 2.9 Shape evolution

An important consideration in using shape spaces is how shape evolution or dynamics may be modeled. If information is available about likely trajectories in a shape space (e.g. from movies), shape dynamics can be generated that mimic real cell shape changes (Johnson *et al.*, 2015b). In the absence of such information, a reasonable assumption is that evolution from one shape towards another should follow a path involving only minimal changes. We can capture this using an energy function to measure how far the shapes along a path of evolution of one shape to another deviates from both of them, as follows:

(5) $$E(x_{1},x_{2})=\frac{\int_{x_{1}}^{x_{2}}(D(f(x),f(x_{1}))+D(f(x_{2}),f(x)))|L\prime (x)|dx}{2\int_{x_{1}}^{x_{2}}|L\prime (x)|dx}$$

where *L*(*x*) is the path, *x* is a point in the latent space, *f*(*x*) is the shape at *x*. $D(f(x),f(x_{1}))$ is the Hausdorff difference between *f*(*x*) and $f(x_{1})$. It is critical to note that the distances are measured between the shapes in the original coordinate space, not the shape space embedding.

For a linear path, the embedding can be approximated with linear interpolation in discrete steps, that is, the form can be simplified and discretized as

(6) $$E(x_{1},x_{2})=\frac{1}{2N}\sum_{i=1}^{N}(D(f({\hat{x}}_{i}),f(x_{1}))+D(f(x_{2}),f({\hat{x}}_{i})))$$

where ${\hat{x}}_{i}=[ix_{1}+(N-i)x_{2}]/N$, and *N* is the number of steps.

We calculated the average energies using 50 000 randomly-chosen pairs of points for 2D datasets, and 5000 for 3D datasets (4950 for HeLa dataset). To facilitate comparisons, a normalized energy was calculated by dividing $E(x_{1},x_{2})$ by $D(f(x_{1}),f(x_{2}))$ for each pair (before averaging).

### 2.10 Joint modeling of cell and nuclear shape

#### 2.10.1 Joint outline PCA and SCA

For these two methods, a ‘separate’ model was defined by forming a reduced dimensionality model separately for cell and nuclear shape. The ‘joint’ model was defined as a model learned simultaneously from both cell and nuclear outline points. To achieve this, the cell and nuclear shapes/preshapes of a given cell were concatenated, and PCA or SCA was performed over the concatenated features to obtain joint low dimensional representations. In both cases, the location (center) and orientation of nuclear shape relative to cell shape were included as features to permit reconstruction with the proper relationship between them. To make a fair comparison of methods, the same total number of dimensions for different methods was used (for separate models, half of the allowed dimensions were used for cell shape and half for nuclear).

#### 2.10.2 Joint autoencoders

For ‘separate’ models, the autoencoder structures described above were used for modeling of cell and nuclear shapes separately. For joint modeling with the Valina autoencoder, two network structures were used. The ‘united’ model used the basic autoencoder structures as before, but the input was indexed images of cell and nuclear shapes, i.e. the pixel values of cell, nuclear and background are 1, 2 and 0, respectively. The softmax cross entropy was used as the loss function for the training. The ‘joint’ model was created as a multi-modal autoencoder (Ngiam *et al.*, 2011). Encoders and decoders were created (separately) for both cell and nuclear shape as before. The outputs of both encoders were then used as inputs for a joint encoding layer with a specified number of latent dimensions. The networks were trained jointly by reducing the sum of the losses.

Because we found that reconstruction using the variational autoencoder had similar yet slightly worse performance than the Valina autoencoder, results for joint modeling with the variational autoencoder are not shown. For joint modeling with structure reference autoencoder, the structures and settings follow those in Johnson *et al.* (2017).

### 2.11 Latent dimension correction across methods

As mentioned above, different methods may take as input raw shapes or shapes normalized for size and/or orientation. For 2D models, all methods except the SCA model have an explicit normalization parameter from alignment of shape, while since the SCA model used preshapes (size normalized), an explicit normalization parameter for size was included. The latent dimension numbers shown in figures and tables do not include these explicit parameters.

For 3D models, the latent dimension was set with the same principle. Autoencoders and SPHARM-RPDM methods were considered to have one normalization parameter because of alignment or image augmentation, while the WSPHARM method had no normalization parameters (and thus 8 or 101 latent dimensions used).

For comparing the joint/separate autoencoders, all the methods had 1 normalization parameter because of joint normalization of rotation.

### 2.12 Implementations

The networks were implemented using TensorLayer (Dong *et al.*, 2017) and Tensorflow (Abadi *et al.*, 2016). The detailed description of the networks used in this paper is described in Supplementary Methods. For the SCA model, the code provided by Lee *et al.* (2016) in github (https://github.com/ccdlcmu/shape_component_analysis_Matlab) was used with some changes in terms of efficiency and adaptation to our analysis pipeline.

## 3 Results

### 3.1 2D cell shape modeling

We began by trying to determine the best available method for modeling 2D cell shapes, as defined by reconstruction error. These methods typically consist of a step to find a representation of shape and a step to reduce the dimensionality of that representation in a model. We could not compare all possible combinations of methods and focused on commonly used approaches. Since as discussed below, outline-based methods performed the best, we did add one combination not previously used: outline extraction followed by an autoencoder. In order to compare methods in a fair manner, we used the same combined number of parameters or dimensions for each. As discussed in the Methods, that number of dimensions included any explicit parameters, such as size that were removed before model training, and any latent dimensions from embedding; for simplicity, we refer to this sum just as the number of latent dimensions throughout. As illustrations of ‘low’ and ‘high’ dimensional models, models were constructed for each method for latent dimensions of 7 and 100; the reconstruction errors are shown in Table 1. For the diffeomorphic model, due to memory constraints and extremely long computing times, only errors for latent dimension 7 for CYTO and HPA CL were calculated. Surprisingly, we found that autoencoders did not perform better than traditional methods. Valina autoencoders generally performed better than other autoencoders. As shown in the table, the outline autoencoder and outline PCA perform similarly for most datasets using 7 dimensions, while outline PCA achieves the best performance across all datasets in 100d. Also, when the dimension was high, the reconstruction errors for all methods were drastically reduced, as expected.

**Table 1.** Reconstruction errors for 2D cell shapes for the five datasets

Dim	Datasets	Outline PCA	SCA	Diffeomorphic	AE	SRAE	VAE	Outline AE
7	CYTO	26.2	37.7	65.0	34.5	66.5	34.7	24.9
	HPA CL	24.2	34.9	54.5	30.2	55.9	29.7	23.1
	H1299	1.22	1.78	–	1.62	1.89	2.49	1.32
	MCF7	8.63	11.6	–	8.93	23.2	9.10	8.13
	SNL	17.3	40.9	–	143.7	106.5	143.3	9.32
100	CYTO	4.20	4.41	–	10.5	34.0	13.5	17.0
	HPA CL	3.76	3.95	–	8.80	15.9	12.4	15.4
	H1299	0.219	0.289	–	1.11	1.78	3.63	1.07
	MCF7	1.38	1.41	–	2.70	5.44	4.55	5.44
	SNL	3.08	3.26	–	4.90	11.5	12.3	7.33
Compute Time		30 min	30 min	∼3.0E4hr	∼ 13hr	18hr	13hr	1hr

*Note*: For diffeomorphic model, only the errors for CYTO and HPA CL datasets in 7d are shown. The computing times are CPU time for outline PCA, SCA and diffeomorphic models and GPU time for autoencoders.

**Fig. 2.**
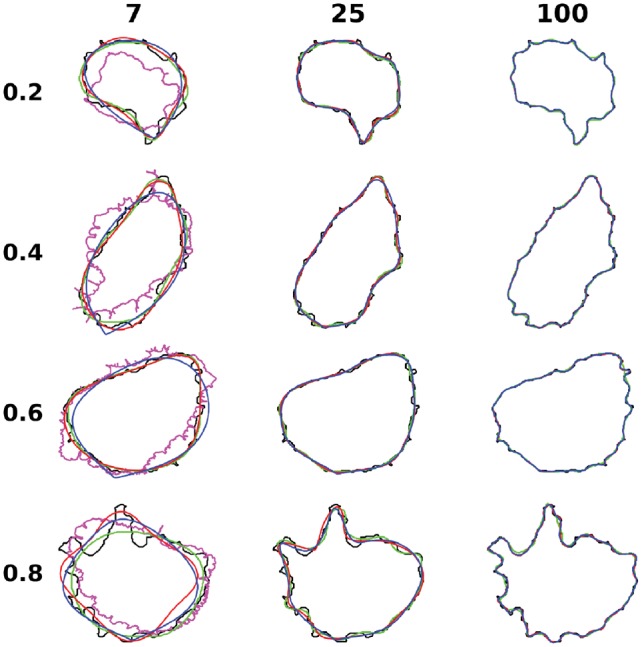
Illustration of representative reconstructions for HPA cell lines dataset. The cells in each row are chosen to represent different quantiles of reconstruction errors from the 100 dimension outline PCA model. The shapes for the original image, diffeomorphic model, outline PCA, SCA and valina autoencoder are represented with black, pink, blue, red and green colors. The columns are for different number of latent dimensions

To illustrate how well the shapes were reconstructed by the different methods, some examples of cell shapes are shown for HPA cell lines in Figure 2. The representative cells shown were chosen using quantiles of errors from the outline PCA model with dimension 100 for the dataset. For low latent dimension for all methods and all quantiles, the reconstructed shapes are quite distinct from the original shapes: the diffeormorphic model reconstructs winding shapes with sharp turns, and its outline matches very poorly with the ground truth, while the other three methods generate very smooth shapes, yet the shapes are still quite different from the original. For the higher latent dimensions, outline PCA, SCA and AE reconstruct better shapes, especially outline PCA and SCA. The reconstructed shapes of outline PCA, SCA and AE have nearly perfect match to the ground truth, especially in dimension 100, while the reconstruction of AE is somewhat smoother than the original images. Similar conclusions can be drawn for the other datasets (Supplementary Figs S6–S9). Interestingly, the pixel-level reconstruction errors (which were defined as the area of non-overlapping region between original and reconstructed images over the area of the original image) as shown in Supplementary Table S1, shows that AE is slightly better than outline PCA and SCA in low dimensional space and has more or less similar performance in high dimensional space. This is because pixel-level reconstruction errors are influenced more by the large number of potential matches inside shapes and do not measure how well the boundaries of shapes are matched. AE can generally cover the main parts of the shape, but it typically not able to match local shape variance (that is, the reconstructed shapes are smoother).

From the above, we can conclude that for 2D cell shapes, for all methods, low dimensional space representations are unable to reconstruct accurate cell shapes, and high dimensional encoding is needed for realistic reconstruction. Outline-based methods are generally better for shape reconstruction, compared with image-based methods. The outline autoencoder works well for low dimensional space but cannot compete with outline PCA in high dimensional space. This is expected because the optimization process in the outline autoencoder tries to find the globally optimal representation by search, while PCA can calculate it directly (the search is harder when the latent dimension is high). Among the various methods, the outline PCA method achieves the best reconstruction performance.

### 3.2 3D cell shape modeling

#### 3.2.1 Comparison of spherical parameterization methods

**Fig. 3.**
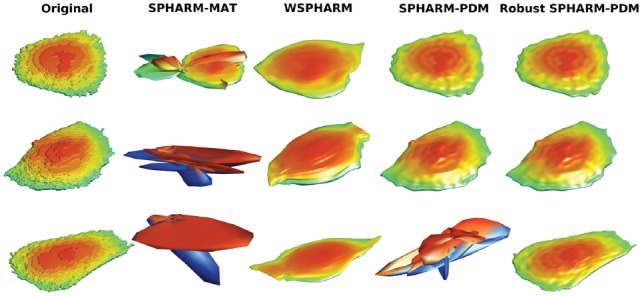
Illustrations of HeLa 3D parameterization reconstruction with different methods. Three cells are randomly picked with the reconstructions for different methods for the same cell in a row

Modeling 2D shapes is generally easy and even simple methods can achieve desirable performance, but this is much less true for 3D shapes. As mentioned before, usually shapes are parameterized as features that capture shape variance. The parameterization is very important and frequently determines the accuracy of models. Before comparing different methods for modeling 3D shapes, we first focused on how the parameterization approach affected performance of one of those methods, SPHARM descriptors. We compared several previously described parameterization methods, with typical results shown in Figure 3, Supplementary Figures S10 and S11.

None of the existing methods worked well for all kinds of cell shapes, whether round cell shapes or complex cell shapes with neurites. However, we found the method in SPHARM-PDM was promising, as it performed well for some cell shapes if the parameterization was successful. However, SPHARM-PDM failed to obtain a parameterization in quite a few cases (Table 2). To get successful parameterization for all shapes (or most shapes), we made several improvements resulting in a method we refer to as SPHARM-RPDM (for Robust SPHARM-PDM). As described in the Methods, these included improvements in the surface preparation, initial parameterization and optimization. As shown in Table 2 and Supplementary Figure S12, none of the cell shapes failed in parameterization with our method, and the improved method needed fewer steps to converge. Though our method is implemented in MATLAB, the convergence times were similar to those in the C++ implementation in SPHARM-PDM. Figure 3, Supplementary Figures S10 and S11 illustrate the successful parameterization performance of our method.

**Table 2.** Performance of spherical parameterization

	Failure rate						Convergence time (s)	
Methods	RPDM			PDM			RPDM	PDM
Dataset	Training	Testing	Overall	Training	Testing	Overall		
Hela	0	0	0	0.225	0.170	0.214	2.20E3	2.71E3
SNL 3D	0	0	0	0.441	0.443	0.441	9.83E2	9.52E2
SNL NR2	0	0	0	0.711	0.725	0.714	1.74E3	1.77E3

*Note*: The left side shows the percentage of cells that have failed parameterizations for SPHARM-RPDM and SPHARM-PDM methods. A parameterization is considered failed if the reconstruction error (for the original descriptor) is greater than 100 pixels. The right side shows the average convergence times.

#### 3.2.2 Comparison of reconstruction errors

Once shape parameterizations (descriptors) were obtained, PCA was used to project the parameterization to a low dimensional latent space (as SCA is a variant of PCA and we found it did not provide significant improvement, we did not include it in further comparisons). For comparison, auteoncoders were used to directly perform dimension reduction from images. To decide which method is the best for 3D shape modeling, and how the dimensionality contributes to the shape reconstruction, shape spaces were built for 7 and 100 latent dimensions; the reconstruction errors are listed in Table 3. For all three datasets, in both 7d and 100d, SPHARM-RPDM performed the best among all methods. For some other spherical harmonic based methods i.e. SPHARM-MAT and SPHARM-PDM, unbounded reconstruction errors from failed parameterizations for some shapes led to very large average errors so these are not shown. The autoencoders did not perform as well as SPHARM-RPDM, and the Valina AE performed better than other AEs.

**Table 3.** Reconstruction error for 3D shapes for the three datasets

Dim	Datasets	SPHARM-RPDM	WSPHARM	Diffeomorphic	AE	SRAE	VAE	O-AE
7	HeLa	8.38	20.4	14.8*	16.2	16.9(17.0)	16.2	40.9
	SNL 3D	8.64	21.4	–	52.7	132.0(42.5)	52.7	57.9
	SNL NR2	12.7	24.6	–	80.1	143.8(51.6)	80.2	73.1
100	HeLa	4.89	20.2	–	7.93	16.9(17.6)	10.4	42.4
	SNL 3D	4.02	16.3	–	7.45	147.3(10.0)	28.4	56.5
	SNL NR2	5.28	21.3	–	8.19	135.0(13.1)	80.1	81.5

*Note*: Same as 2D datasets, we only show the results for 7 dimensions for HeLa 3D dataset for diffeomorphic model. In the parenthesis, we show the reconstruction errors after filtering out isolated voxels in the reconstruction. *The reconstruction error for the diffeomorphic model was calculated with only a subset of the shapes because it failed for many of them.

**Fig. 4.**
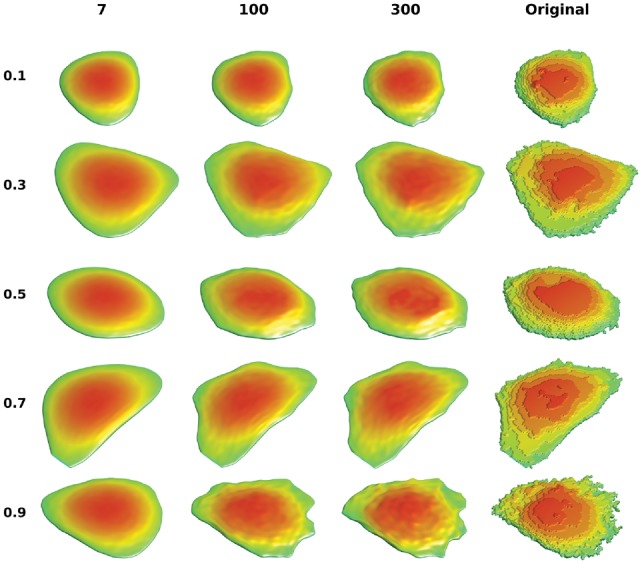
Illustration of HeLa 3D reconstruction with SPHARM-RPDM method. The cells are chosen based on the quantiles of reconstruction errors in latent dimension 300, which are listed in the left side. The reconstructions of different latent dimensions are shown with same cell in the same row, along with the ground truth

The reconstructions of representative shapes across different latent dimensions are shown in Figure 4 and Supplementary Figures S13–S17. For all methods, similar to our findings for 2D, we observe that low dimensional encodings can only reconstruct smooth shapes. As the dimension increases, more shape variance can be captured. However, even in high dimensional space, AE are not able to capture fine shape variances.

### 3.3 Shape evolution

**Fig. 5.**
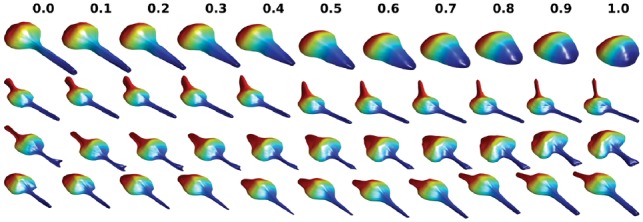
Illustrations of shape evolutions for SNL 3D dataset. Four pairs of cells are randomly selected. The source, target and intermediate shapes in the linear path are shown, with the title showing the distance to the source. The source and target are labeled as 0 and 1

An important application of shape space methods is to model shape evolution. This is relevant to studying cell dynamics, e.g. during cell movement and cell growth. Neuron differentiation from an approximately round cell to a neuron with neurites is a particular example. As described in the Methods section, interpolation in the shape space can be applied to find intermediate shapes between source and target shapes. Some examples are shown in Figure 5, Supplementary Figures S18 and S19. These figures illustrate that SPHARM-RPDM performs well for simulating this transition. The results are especially noteworthy for SNL 3D shapes, in that it simulates a process of neurite growth, even though no knowledge of how a neurite grows is used in the model.

These simulations assume that no information is available about the likely changes that might occur between two specific shapes (or even if they are likely to occur at all). In this case, a reasonable assumption is that the evolution between two shapes takes place along a path that minimizes the total difference between the intermediate shapes and the starting and ending shape. Depending on the method used for shape space construction, finding this path is typically quite expensive. In diffeomorphic methods, extensive computation is done to find the minimum energy path, and hence the distance, between all pairs of shapes before the shape space is constructed, and synthesizing along this path is therefore inexpensive. In other methods that are faster, the minimal energy path would need to be found by search after the construction of the shape space. Thus, minimum energy evolution is expensive either way (especially in high dimensions). As a practical consideration, we therefore investigated whether simply interpolating along a linear path would give a reasonably low energy. Using an evolution energy measure based on Hausdorff distance as described in the Methods, the results of linear interpolations in shape space are shown for SPHARM-RPDM and AE in Table 4. For each method, the evolution energy is smaller when the latent dimension is high, consistent with our previous observation that intermediate shapes in higher dimensions are more like the source and target shapes. Again our method (SPHARM-RPDM) performs better than Valina AE for both low and high dimensional space in terms of shape evolution.

**Table 4.** Energies of shape evolution

Latent dimensions		7		100	
Methods Dataset		AE	Outline PCA/RPDM	AE	Outline PCA/RPDM
2D	CYTO	0.714	0.604	0.556	0.539
	HPA CL	0.698	0.599	0.551	0.536
	H1299	0.674	0.586	0.619	0.521
	MCF7	0.656	0.620	0.549	0.545
	SNL	1.61	0.565	0.527	0.534
3D	HeLa	0.869	0.586	0.875	0.545
	SNL 3D	1.35	0.576	0.588	0.538
	SNL NR2	1.80	0.621	0.640	0.555

*Note*: The energies for dimensions 7 and 100 for AE and outline PCA (2D) and SPHARM-RPDM (3D) are shown. The normalized energies by the Hausdorff distance between the source and target are shown in the table (the optimal value is 0.5). Smaller energies mean more efficient transformations.

### 3.4 Joint modeling of cell and nuclear shape

**Fig. 6.**
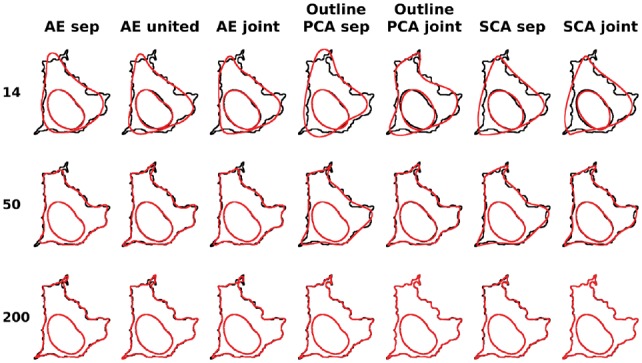
Illustrations of joint modeling of cell and nuclear shapes for different methods for CYTO dataset. Here we choose a cell in the quantile of 0.65 for the joint outline errors for PCA joint models in 200d. The original shape is shown in black and the reconstruction is shown in red. This cell is shown across different methods and different latent dimensions, which are indicated in the title and the Y-axis, respectively

The relationship between cell and nuclear shape is also an important aspect that may be involved in cellular processes. Previous work has shown that there are dependency relationship between the two (Johnson *et al.*, 2015b) that may be disrupted under certain conditions. We therefore next evaluated the performance of various methods on the task of modeling both cell and nuclear shape. One potential criterion for reconstruction error for joint modeling is to apply Hausdorff distance for both cell and nuclear shapes, however, in this case, the errors for nuclear shapes will be missed because of the dominance of cell shape errors. Therefore, we calculated the reconstruction errors for cell and nuclear shapes separately and averaged them as the joint error. Reconstruction errors for both separate and joint models are shown in Tables 5 and 6, with the errors for cell and nuclear shapes shown in Supplementary Tables S2 and S3. Some example shapes are illustrated in Figure 6 and Supplementary Figure S20 for 2D and 3D, respectively. As seen in the tables, for each method, the differences of the reconstruction accuracy for joint modeling and separate modeling are small for the same method in the same latent dimension. Again among all methods, outline PCA for 2D and our method for 3D have the best performances either with joint or separate modeling. As shown in Supplementary Tables S2 and S3, for outline PCA methods, joint model errors for cell shape decrease while the errors for nuclear shape increase, compared with the separate models, indicating that greater overall weight was put on the cell shape in the joint models. However, here we should stress that the success of separate modeling does not imply that there is no relationship between the two components (that relationship is captured by explicit features). It simply means that there is no advantage to taking that relationship into account when learning the component shape models themselves.

**Table 5.** Reconstruction errors of 2D joint modeling

		Outline PCA		SCA		AE			SRAE	
Dim	Datasets	sep	joint	sep	joint	sep	united	joint	sep	joint
14	CYTO	16.1	16.6	20.5	20.6	20.2	17.6	20.0	36.2	55.8
	HPA CL	14.9	15.4	19.2	19.3	17.9	16.0	17.6	30.7	32.7
	H1299	1.20	1.35	1.70	1.68	5.43	1.37	1.43	5.56	1.90
	MCF7	5.25	5.21	6.02	6.04	5.32	4.88	5.22	12.4	6.43
200	CYTO	2.43	2.03	2.54	2.12	6.16	5.47	5.95	17.9	14.2
	HPA CL	2.20	1.86	2.31	1.95	5.30	4.87	5.08	8.82	10.7
	H1299	0.168	0.194	0.199	0.215	7.77	1.01	4.08	8.34	2.21
	MCF7	0.918	0.928	1.02	0.939	1.92	2.00	1.99	3.29	4.60

*Note*: The table shows two latent dimensions 14 and 200 for the four 2D datasets for four representative methods. ‘sep’, ‘joint’ and ‘united’ means separate models, joint models and united models for corresponding methods as described in Methods, respectively.

**Table 6.** Reconstruction errors for the joint modeling of HeLa 3D dataset

	SPHARM-RPDM		AE			SRAE	
Dim	sep	joint	sep	united	joint	sep	joint
14	5.78	5.72	9.72	56.0	8.48	10.0	–
200	3.45	3.42	5.12	5.56	6.02	9.63	–

*Note*: The errors for the three representative methods with dimensions 14 and 200 are shown. Errors of SRAE separate models are unavailable because SRAE nuclear model fails in reconstruction.

## 4 Discussion

We have compared various approaches for modeling of cell and/or nuclear shape. For 2D cell shapes, in low dimensional space, all of these methods reconstruct overly-smooth shapes that are not realistic. When increasing latent dimension, these methods can all achieve better reconstruction performances, however, deep autoencoders are not as good as outline PCA and SCA methods, which can well preserve local variance and thus reconstruct shapes nearly perfectly. For 3D cell/nuclear shapes, we describe an improved method that achieves the best performance when compared to previous methods, and is especially useful for modeling complex cell shapes. Our method improves the robustness of the mapping of the original cell shape to a sphere, and works well even for highly non-spherical shapes. We also showed, as may be expected, that these methods (outline PCA for 2D/SPHARM-RPDM PCA for 3D) also perform better at shape evolution, with computationally efficient, linear interpolation in the shape space coming close to the minimum deformation energy.

Therefore, we suggest that if shape representation/evolution is the major consideration for the modeling of cell shapes, these methods would be the best choice, due to their computational efficiency and strong performance. Deep learning methods typically require a lot more computing resources and more training data (of course, deep learning methods are continuously evolving and this may change). Moreover, we recommend that outline/surface-based shape models are a better choice than whole shape-based methods. However, our results do not necessarily mean that other methods are inferior in all aspects of cell analysis. As shown previously, diffeomorphic models work well for mapping protein distributions inside cells (Roybal *et al.*, 2016). In addition, supervised deep learning methods often significantly outperform traditional methods for classification tasks (in which detailed cell shape information may not be needed). Thus, careful consideration should be given based on the specific task when working with cell images.

It should be noted that depictions of cell shape changes such as those in Figure 5 are not necessarily accurate. In the absence of any additional information, however, they represent the best prediction that can be made. When available, movies can be used to calculate vector fields in the shape space that may give more accurate cell shape changes (Johnson *et al.*, 2015b). Even so, they can only be accurate up to the temporal resolution of the movies.

We have focused here on the task of accurate cell and nuclear shape reconstruction from models. Such models have two major uses. The first is to provide a more complete lower dimensional representation that arbitrarily chosen descriptive features (a goal often referred to as compression). Such a representation is useful for improving comparison of different populations and for clustering. It is also useful to provide a basis for learning the dependency of other cell components upon cell and/or nuclear shape, or for removing that dependency to enable comparison of subcellular distributions for cells that different in shape (Zhao and Murphy, 2007). Of course the second major use is for the synthesis of new cell and nuclear shapes. These are useful in themselves for illustrative or educational purposes, especially for the production of movies that show learned or predicted shape dynamics. They can also be used to produce well-characterized geometries (either static or dynamic) for simulations of cell biochemistry or behavior that may be better than geometries derived directly from individual cell images or movies. This is in part because models learned from many cells may be able to fill in some spatial frequencies or structures not contained in single acquired images. Lastly, but perhaps most importantly, generative models can combine information from different image sets to produce images or movies representing components that were not imaged in the same cell (Murphy, 2012). This can be done by separately learning models of the spatiotemporal dependencies of components upon a reference structure (such as cell shape) and then predicting a joint distribution by combining the models (Johnson *et al.*, 2015a, 2017).

## Supplementary Material

bty983_Supplementary_Data

## References

[bty983-B1] Abadi M. et al (2016) Tensorflow: a system for large-scale machine learning. OSDI, 16, 265–283.

[bty983-B2] Bay H. et al (2008) Speeded-up robust features (surf). Comput. Vis. Image Understand., 110, 346–359.

[bty983-B3] Beg M.F. et al (2005) Computing large deformation metric mappings via geodesic flows of diffeomorphisms. Int. J. Comput. Vis., 61, 139–157.

[bty983-B4] Brechbühler C. et al (1995) Parametrization of closed surfaces for 3-d shape description. Comput. Vis. Image Understand., 61, 154–170.

[bty983-B5] Caie P.D. et al (2010) High-content phenotypic profiling of drug response signatures across distinct cancer cells. Mol. Cancer Therap., 9, 1913–1926. 20530715 10.1158/1535-7163.MCT-09-1148

[bty983-B6] Chung M.K. et al (2007) Weighted fourier series representation and its application to quantifying the amount of gray matter. IEEE Trans. Med. Imag., 26, 566–581. 10.1109/TMI.2007.892519 17427743

[bty983-B7] Dong H. et al (2017) Tensorlayer: a versatile library for efficient deep learning development. In: *Proceedings of the 2017 ACM on Multimedia Conference*, ACM, Mountain View, CA, pp. 1201–1204.

[bty983-B8] Dryden I.L., Mardia K.V. (2016) Statistical Shape Analysis: With Applications in R. John Wiley & Sons, Chichester.

[bty983-B9] Du C.-J. et al (2013) 3d time series analysis of cell shape using laplacian approaches. BMC Bioinformatics, 14, 296. 24090312 10.1186/1471-2105-14-296 PMC3871028

[bty983-B10] Ducroz C. et al (2012) Characterization of cell shape and deformation in 3d using spherical harmonics In: Biomedical Imaging (ISBI), 2012 9th IEEE International Symposium on IEEE, Barcelona, Spain, pp. 848–851.

[bty983-B11] Goodfellow I. et al (2014) Generative adversarial nets In: Advances in Neural Information Processing Systems, Montréal, Canada, pp. 2672–2680.

[bty983-B12] He K. et al (2016) Deep residual learning for image recognition. In *Proceedings of the IEEE Conference on Computer Vision and Pattern Recognition*, Las Vegas, NV, pp. 770–778.

[bty983-B13] Hinton G.E., Salakhutdinov R.R. (2006) Reducing the dimensionality of data with neural networks. Science, 313, 504–507. 16873662 10.1126/science.1127647

[bty983-B14] Johnson G.R. et al (2015a) Automated learning of subcellular variation among punctate protein patterns and a generative model of their relation to microtubules. PLoS Comput. Biol., 11, 1–17. 10.1371/journal.pcbi.1004614 PMC4704559 26624011

[bty983-B15] Johnson G.R. et al (2015b) Joint modeling of cell and nuclear shape variation. Mol. Biol. Cell, 26, 4046–4056. 26354424 10.1091/mbc.E15-06-0370 PMC4710235

[bty983-B16] Johnson G.R. et al (2017) Generative modeling with conditional autoencoders: Building an integrated cell. https://arxiv.org/help/faq/references.

[bty983-B17] Kazhdan M. et al (2003) Rotation invariant spherical harmonic representation of 3D shape descriptors. In: *Symposium on Geometry Processing*, Aachen, Germany, pp. 156–164.

[bty983-B18] Kingma D.P., Welling M. (2013) Auto-encoding variational bayes. *arXiv: 1312.6114 [stat.ML].*

[bty983-B19] Lee H.-C. et al (2016) Shape component analysis: structure-preserving dimension reduction on biological shape spaces. Bioinformatics, 32, 755–763. 26543176 10.1093/bioinformatics/btv648

[bty983-B20] Lowe D.G. (2004) Distinctive image features from scale-invariant keypoints. Int. J. Comput. Vis., 60, 91–110.

[bty983-B21] Makhzani A. et al (2015) Adversarial autoencoders. *arXiv: 1511.05644 [cs.LG].*

[bty983-B22] Masci J. et al (2011) Stacked convolutional auto-encoders for hierarchical feature extraction. In: *International Conference on Artificial Neural Networks*, Espoo, Finland, pp. 52–59.

[bty983-B23] Murphy R.F. (2012) Chapter 7 – cellorganizer: image-derived models of subcellular organization and protein distribution In: Asthagiri A.R., Arkin A.P. (eds) Computational Methods in Cell Biology, volume 110 of Methods in Cell Biology, Academic Press, Cambridge, MA, pp. 179–193. 10.1016/B978-0-12-388403-9.00007-2 PMC4107418 22482949

[bty983-B24] Ngiam J. et al (2011) Multimodal deep learning. In *Proceedings of the 28th International Conference on Machine Learning (ICML-11)*, Bellevue, WA, pp. 689–696.

[bty983-B25] Osokin A. et al (2017) Gans for biological image synthesis. In: *2017 IEEE International Conference on Computer Vision (ICCV)*, IEEE, Venice, Italy, pp. 2252–2261.

[bty983-B26] Pincus Z., Theriot J. (2007) Comparison of quantitative methods for cell-shape analysis. J. Microsc., 227, (2) 140–156. 17845709 10.1111/j.1365-2818.2007.01799.x

[bty983-B27] Press W.H. et al (2007) Numerical Recipes 3rd edition: The Art of Scientific Computing. Cambridge University Press, Cambridge, England.

[bty983-B28] Rohde G.K. et al (2008) Deformation-based nuclear morphometry: capturing nuclear shape variation in hela cells. Cytometry A, 73, 341–350. 18163487 10.1002/cyto.a.20506

[bty983-B29] Roybal K.T. et al (2016) Computational spatiotemporal analysis identifies wave2 and cofilin as joint regulators of costimulation-mediated t cell actin dynamics. Sci. Signal., 9, rs3–rs3. 27095595 10.1126/scisignal.aad4149 PMC4871116

[bty983-B30] Rumelhart D.E. et al (1986) Learning representations by back-propagating errors. Nature, 323, 533.

[bty983-B31] Shen L., Makedon F. (2006) Spherical mapping for processing of 3d closed surfaces. Image Vis. Comput., 24, 743–761.

[bty983-B32] Shen L. et al (2009) Modeling three-dimensional morphological structures using spherical harmonics. Evolution, 63, 1003–1016. 19154365 10.1111/j.1558-5646.2008.00557.x PMC2936781

[bty983-B33] Sigal A. et al (2006) Dynamic proteomics in individual human cells uncovers widespread cell-cycle dependence of nuclear proteins. Nat. Methods, 3, 525. 16791210 10.1038/nmeth892

[bty983-B34] Sigal A. et al (2007) Generation of a fluorescently labeled endogenous protein library in living human cells. Nat. Protoc., 2, 1515. 17571059 10.1038/nprot.2007.197

[bty983-B35] Styner M. et al (2006) Framework for the statistical shape analysis of brain structures using spharm-pdm. Insight J., 1071, 242. PMC3062073 21941375

[bty983-B36] Tangelder J.W., Veltkamp R.C. (2008) A survey of content based 3d shape retrieval methods. Multimedia Tools Appl., 39, 441–471.

[bty983-B37] Velliste M., Murphy R.F. (2002) Automated determination of protein subcellular locations from 3d fluorescence microscope images. In: *Proceedings 2002 IEEE International Symposium on Biomedical Imaging, 2002*, IEEE, Washington, DC, pp. 867–870.

[bty983-B38] Vincent L., Soille P. (1991) Watersheds in digital spaces: an efficient algorithm based on immersion simulations. IEEE Trans. Pattern Anal. Mach. Intell., 583–598.

[bty983-B39] Zhao T., Murphy R.F. (2007) Automated learning of generative models for subcellular location: building blocks for systems biology. Cytometry A, 71A, 978–990. 10.1002/cyto.a.20487 17972315

