# Supplementary material for: Evaluation of methods for generative modeling of cell and nuclear shape
Source: Bioinformatics. 2018 Dec 7;35(14):2475–85. doi: 10.1093/bioinformatics/bty983 (PMC6612826; doi:10.1093/bioinformatics/bty983)
Supplement: bty983_Supplementary_Data [file bty983_supplementary_data.pdf]

Supplement to

Evaluation of methods for generative modeling of cell and nuclear shape

Xiongtao Ruan
Computational Biology Department, School of Computer Science
Carnegie Mellon University Pittsburgh, PA 15213

Robert F. Murphy
Computational Biology Department, School of Computer Science
Carnegie Mellon University Pittsburgh, PA 15213
murphy@cmu.edu

Supplementary methods

1 Simulation process of simulated cells

1.1 Detailed simulation process of SNL cells

The simulation process was:

- Sampling of cell body. First, we sample the cell body as the combination of two half ellipses, using radial coordinates. The two half ellipses share the same semiminor axis as $b \sim U(10, 20)$, and the two semimajor axis a_1 and a_2 also have distribution uniform distribution $U(10, 20)$. Then a Gaussian random noise with standard deviation 0.1 are added into the coordinates. In the next step, we morph and smooth the cell body using spline smoothing method. We randomly sample the proportion of points as control points with distribution $U(0.01, 0.20)$. After randomly choosing control points, we allow the points to randomly move along it's normal direction, and the step size follows $U(1, 1.5) * N(0, 0.1)$, which is the combination of uniform and Normal distribution. Then the moved control points are used for spline smoothing. At last, we add some Gaussian noises to the shape with standard deviation 0.05.
- Sampling of Neurites. The neurite number N can be either fixed or randomly sampled following a truncated Poisson distribution with $\lambda = 1$ for a given range of allowed neurite number (0-2 for SNL 3D center slice). First we sample the total length of neurites following $U(20N, 25N)$. To assign proportions of length for the two neurites, we use a truncated Dirichlet distribution $Dirichlet(2, 1)$ and force the smaller proportion to be larger than 0.2.
- Sampling of branching. Then, we may decide whether a relatively long neurite, which is longer than 5, could generate branches, with a probability 0.4. We assign the weight of length using truncated Dirichlet distribution $Dirichlet(1, 1, 1)$ with any weight should be larger than 0.05. We assign the largest weight to the main part connecting cell body. Then we sample the angles for the two branches with the major axis of the main part. $\theta_1 = 0$ with probability 0.5, and $\theta_1 \sim U(-0.45\pi, 0.45\pi)$ with probability 0.5. If $\theta_1 \geq 0$, $\theta_2 \sim U(-0.225\pi, 0.45\pi)$; if $\theta_1 < 0$, $\theta_2 \sim U(-0.225\pi, 0.45\pi)$. In this case, we can ensure the two branches are in the two sides of the major axis.

- Sampling of thickness of neurites. For each neurite, if there is no branching, the thickness T_0 at the end point connecting the cell body is sampled with distribution $U(2,3)$, and the other end point T_1 is sampled as the maximum of $U(0.4T_0, T_0)$ and $T_0 - 0.02L + U(0, 0.05)$, where L is the length of the neurite. If there is branching, $L_0 \sim U(2,3)$, the thickness in the branching point $T_2 \sim \max(U(0.4T_0, T_0), L_0 - 0.02L_1 + U(0, 0.05))$. the thickness in the two end points of the branches follows $T_3 \sim \max(U(0.4T_2, T_2), L_0 - 0.02L_2 + U(0, 0.05))$ and $T_4 \sim U(0.4T_2, T_2)$, where L_2 is the length of the first branch.
- Connecting neurites and cell body. We first put two neurites to angle 0 and π relative to the cell body, then we allow some small perturbations of the angles with truncated normal distribution $N_{[-0.22,0.22]}(0, 0.125^2) * 2\pi$. The truncated normal distribution here means that only the random variable between -0.22 and 0.22 from the distribution will be accepted. After this step, we connect the neurites and cell body by removing some points in the cell body while connecting with the end points of neurites, so that it is a valid ordered array for the outline.
- Post-processing. Lastly, We introduce random rotation and variation in size. The rotation angle follows $U(0, 2\pi)$, and the scale factor follows $N(0.5, 0.5^2)$. We translate the coordinates by $[256.5, 256.5]$ and convert that to a binary image with size $512X512$, so that the shapes are centered.

1.2 Detailed simulation process for SNL (NR2) 3D datasets

The simulation process was:

- Sampling of central slice. A central slice is sampled from the SNL 2d simulator, as described above. For SNL 3D dataset, the neurite number ranges randomly from 0-2. For SNL 3D dataset, the neurite number is fixed as 2.
- Sampling of upper slices. Starting from the central slice, image erosion is applied to the current slice, with a disk kernel with a random kernel size. The kernel size is randomly sample between 1-3 with probabilities $0.45,\ 0.35,\ 0.2$, for the first five slices, and between 2-5 with probabilities $0.3125,\ 0.2625,\ 0.2625,\ 0.1625$, after the first five slices. The erosion is stopped until the area of the current slice is equal or smaller than 500 pixels.
- Sampling of bottom slices. Similar as sampling for upper slices, starting from the central slice, image erosion or dilation is applied to the current slice, with a disk kernel with a random kernel size. The probability of erosion and dilation are 0.5 and 0.5. The kernel size is randomly sample between 0 - 3 with probabilities $0.25,\ 0.45,\ 0.2,\ 0.1$, for the first ten slices, and between 0-4 with probabilities $0.25,\ 0.25,\ 0.2,\ 0.2,\ 0.2$, after the first ten slices. The erosion or dilation is stopped until the ratio of the area of the current slice to that of the central slice is between 0.85 and 1.15. After that, the 3D image is stacked with bottom slices, central slice and upper slices.
- Adding random noise. The positions of seeds are randomly sampled with probability 0.005 in the image. Then image dilation with sphere kernel with size 1 is applied to the seeds to get the random noise image. The same process is repeated to get another random noise image. Then the first noise image is added to the cell image, followed by the subtraction of the second noise image. Then the cell image with noise is binarized by threshold of positive values. Only the largest connected component is kept and holes are filled.
- Image cropping and resizing. The cell image is cropped to slice number 24 by keeping middle 24 slices. Finally the image is resized to the size $256 \times 256 \times 24$.

2 Deep networks

2.1 Structures of 2D networks

2.1.1 Definition of residual blocks

In the main paper we briefly described the network architecture without introduce residual network blocks. Here we show the detailed structure for residual network blocks for both encoder and decoder networks in following three tables.

Layer name	Layer type	Parent Layer	Tensor size	Miscellaneous
B1	Input	None	$2m \times 2m \times n_0$	
B2	Conv2d	B1	$m \times m \times n$	downsample via subsampling 2×2
B3	BatchNorm	B2	$m \times m \times n$	contains ReLu activation
B4	Conv2d	B3	$m \times m \times n$	
B5	BatchNorm	B4	$m \times m \times n$	contains by ReLu activation
B6	Conv2d	B5	$m \times m \times n$	
B7	Merge	B6, B2	$m \times m \times n$	sum the output of two layers
B8	Output	B7	$m \times m \times n$	

Residual block type 1 in the encoder network (EB1). Here B1-B8 represent layer names to distinguish these layers here (not same as the implementations); Layer type uses names quit similar to those used in TensorLayer. Input and Output are not actual layers, but just illustrate the start and end of the flow. Parent Layer means the layer that the current layer connects. Tensor size represents the output size. Here $2m \times 2m$ are abstract input data size, n_0 is the filter number of previous block and n is the filter number for current block. Basically the block down sample the image size 2×2, changes filter size from n_0 to n. Miscellaneous column shows some detailed explanations.

Layer name	Layer type	Parent Layer	Tensor size	Miscellaneous
B1	Input	None	$(m+2h) \times (m+2h) \times n_0$	
B2	Conv2d	B1	$m \times m \times n$	No downsampling and no padding.
B3	BatchNorm	B2	$m \times m \times n$	contains ReLu activation
B4	Conv2d	B3	$m \times m \times n$	
B5	BatchNorm	B4	$m \times m \times n$	contains by ReLu activation
B6	Conv2d	B5	$m \times m \times n$	
B7	Merge	B6, B2	$m \times m \times n$	sum the output of two layers
B8	Output	B7	$m \times m \times n$	

Residual block type 2 in the encoder network (EB2). The basic structure is similar as EB1, except that there is no downsampling and no padding in B2 layer, in order to change the image size. Here $(m+2h) \times (m+2h)$ are abstract input data size, where m is the output image size for the block, $2h$ is the size that is reduced in convolution, $h = \lceil (k-1)/2 \rceil$, where k is the filter size. n_0 is the filter size of previous block and n is the filter number for current block. Miscellaneous column shows some detailed explanations.

Layer name	Layer type	Parent Layer	Tensor size	Miscellaneous
B1	Input	None	$m \times m \times n_0$	
B2	Conv2d	B1	$m \times m \times n$	
B3	BatchNorm	B2	$m \times m \times n$	contains ReLu activation
B4	Conv2d	B3	$m \times m \times n$	
B5	BatchNorm	B4	$m \times m \times n$	contains by ReLu activation
B6	Conv2d	B5	$m \times m \times n$	
B7	Merge	B6, B2	$m \times m \times n$	sum the output of two layers
B8	UpSampling	B7	$2m \times 2m \times n$	upsample 2×2
B9	Output	B8	$2m \times 2m \times n$	

Residual block in the decoder network (DB1). The table has almost the same interpretation as last table for encoder network. There are some differences: for layer B2, there is no down sampling in the convolutional layer, instead it has an additional layer after layer B7 for upsampling to change the image size from $m \times m$ to $2m \times 2m$.

2.1.2 Network Structure for Valina autoencoders for CYTO, HPA CL, SNL datasets

These three datasets share the same network structures, which is defined as follows:

Block	Input	EB1	EB1	EB1	EB1	EB1	EB1	EB1	Flatten	Fully Connected
Filter size	-	(3, 3, 2)	(3, 3, 4)	(3, 3, 8)	(3, 3, 16)	(3, 3, 32)	(3, 3, 64)	(3, 3, 128)		1
Output size	(512, 512, 1)	(256, 256, 2)	(128, 128, 4)	(64, 64, 8)	(32, 32, 16)	(16, 16, 32)	(8, 8, 64)	(4, 4, 128)	2048×1	$l \times 1$

Encoder network for CYTO, HPA CL and SNL datasets for Valina autoencoders. l is the desired latent dimension. The input image size is 512×512. For the size of filters, the first two numbers are the width and height of the filter and the third one is for the filter number. The output size has the same format, that is, the numbers are width, height and number of channels. The layers/blocks are ordered from left to right in the network (below the same).

Using the output of encoder network as input, the decoder network is defined as:

Block	Input	Fully Connected	Reshape	DB1	DB1	DB1	DB1	DB1	DB1	DB1	Conv2d
Filter size	-	2048	-	(3, 3, 128)	(3, 3, 64)	(3, 3, 32)	(3, 3, 16)	(3, 3, 8)	(3, 3, 4)	(3, 3, 2)	(3, 3, 1)
Output size	$l \times 1$	2048×1	(4, 4, 128)	(4, 4, 128)	(8, 8, 64)	(16, 16, 32)	(32, 32, 16)	(64, 64, 8)	(128, 128, 4)	(256, 256, 2)	(512, 512, 1)

Decoder network for CYTO, HPA CL and SNL datasets for Valina autoencoders. l is the desired latent dimension.

2.1.3 Network Structure for Valina autoencoders for H1299 datasets

Similar as above, the encoder and decoder networks are defined as:

Block	Input	EB1	EB1	EB1	EB1	EB1	EB2	Flatten	Fully Connected
Filter size	-	(3, 3, 16)	(3, 3, 32)	(3, 3, 64)	(3, 3, 128)	(3, 3, 256)	(3, 3, 512)		1
Output size	(80, 80, 1)	(40, 40, 16)	(20, 20, 32)	(10, 10, 64)	(5, 5, 128)	(3, 3, 256)	(1, 1, 512)	512×1	$l \times 1$

Encoder network for H1299 dataset for Valina autoencoders. l is the desired latent dimension. The input image size is 80×80. For the size of filters, the first two numbers are the width and height of the filter and the third one is for the filter number. The output size has the same format, that is, the numbers are width, height and number of channels.

Block	Input	Fully Connected	Reshape	DB1	DB1	PadLayer	DB1	DB1	DB1	DB1	Conv2d
Filter size	-	512	-	(3, 3, 128)	(3, 3, 64)	(3, 3, 32)	(3, 3, 16)	(3, 3, 8)	(3, 3, 4)	(3, 3, 2)	(3, 3, 1)
Output size	$l \times 1$	512×1	(1, 1, 512)	(2, 2, 512)	(4, 4, 256)	(5, 5, 256)	(10, 10, 128)	(20, 20, 64)	(40, 40, 32)	(80, 80, 16)	(80, 80, 1)

Decoder network for H1299 dataset for Valina autoencoders. l is the desired latent dimension.

2.1.4 Network Structure for Valina autoencoders for MCF7 datasets

Similar as above, the encoder and decoder networks are defined as:

Block	Input	EB1	EB1	EB1	EB1	EB1	EB1	EB2	Flatten	Fully Connected
Filter size	-	(3, 3, 4)	(3, 3, 8)	(3, 3, 16)	(3, 3, 32)	(3, 3, 64)	(3, 3, 128)	(3, 3, 512)		1
Output size	(160, 160, 1)	(80, 80, 4)	(40, 40, 8)	(20, 20, 16)	(10, 10, 32)	(5, 5, 64)	(3, 3, 128)	(1, 1, 512)	512×1	$l \times 1$

Encoder network for MCF7 dataset for Valina autoencoders. l is the desired latent dimension. The input image size is 160×160. For the size of filters, the first two numbers are the width and height of the filter and the third one is for the filter number. The output size has the same format, that is, the numbers are width, height and number of channels.

Block	Input	Fully Connected	Reshape	DB1	DB1	PadLayer	DB1	DB1	DB1	DB1	DB1	Conv2d
Filter size	-	512	-	(3, 3, 512)	(3, 3, 128)	(3, 3, 64)	(3, 3, 32)	(3, 3, 16)	(3, 3, 8)	(3, 3, 4)	(3, 3, 2)	(3, 3, 1)
Output size	$l \times 1$	512×1	(1, 1, 512)	(2, 2, 512)	(4, 4, 128)	(5, 5, 128)	(10, 10, 64)	(20, 20, 32)	(40, 40, 16)	(80, 80, 8)	(160, 160, 4)	(160, 160, 1)

Decoder network for MCF7 dataset for Valina autoencoders. l is the desired latent dimension.

2.2 Structures of 3D networks

2.2.1 Definition of residual blocks

In the main paper we briefly described the network architecture without introduce residual network blocks. Here we show the detailed structure for residual network blocks for both encoder and decoder networks in the following three tables.

Layer name	Layer type	Parent Layer	Tensor size	Miscellaneous
B1	Input	None	$am \times am \times bt \times n_0$	
B2	Conv3d	B1	$m \times m \times t \times n$	subsampling with size (a, a, b).
B3	BatchNorm	B2	$m \times m \times t \times n$	contains ReLu activation
B4	Conv3d	B3	$m \times m \times t \times n$	
B5	BatchNorm	B4	$m \times m \times t \times n$	contains by ReLu activation
B6	Conv3d	B5	$m \times m \times t \times n$	
B7	Merge	B6, B2	$m \times m \times t \times n$	sum the output of two layers
B8	Output	B7	$m \times m \times t \times n$	

Residual block in the 3D encoder network (EB3D). Here B1-B8 represent layer names to distinguish these layers here (not same as the implementations); Layer type uses names quit similar to those used in TensorLayer. Input and Output are not actual layers, but just illustrate the start and end of the flow. Parent Layer means the layer that the current layer connects. Tensor size represents the output size. Here $am \times am \times bt$ are abstract input data size, n_0 is the filter number of previous block and n is the filter number for current block. Basically the block downsamples the image size $a \times a \times b$, changes filter size from n_0 to n. Miscellaneous column shows some detailed explanations.

Layer name	Layer type	Parent Layer	Tensor size	Miscellaneous
B1	Input	None	$m \times m \times t \times n_0$	
B2	Conv3d	B1	$m \times m \times t \times n$	
B3	BatchNorm	B2	$m \times m \times t \times n$	contains ReLu activation
B4	Conv3d	B3	$m \times m \times t \times n$	
B5	BatchNorm	B4	$m \times m \times t \times n$	contains by ReLu activation
B6	Conv3d	B5	$m \times m \times t \times n$	
B7	Merge	B6, B2	$m \times m \times n$	sum the output of two layers
B8	UpSampling	B7	$am \times am \times bt \times n$	upsample $a \times a \times b$
B9	Output	B8	$am \times am \times bt \times n$	

Residual block in the 3D decoder network (DB3D). The table has almost the same interpretation as last table for encoder network. There are some differences: for layer B2, there is no down sampling in the convolutional layer, instead it has an additional layer after layer B7 for upsampling to change the image size from $m \times m \times t$ to $am \times am \times bt$.

2.2.2 Network Structure for Valina autoencoders for HeLa, SNL 3D and SNL NR2 3D datasets

These three datasets share the same network structure, which is defined as follows:

Block	Input	EB3D	EB3D	EB3D	EB3D	EB3D	EB3D	EB3D	Flatten	Fully Connected
Filter size	-	(3, 3, 3, 2)	(3, 3, 3, 4)	(3, 3, 3, 8)	(3, 3, 3, 16)	(3, 3, 3, 32)	(3, 3, 3, 64)	(3, 3, 3, 128)		l
(a, b)	-	(2, 2)	(2, 2)	(2, 2)	(2, 1)	(2, 1)	(2, 1)	(2, 1)		
Output size	(256, 256, 24, 1)	(128, 128, 12, 4)	(64, 64, 6, 8)	(32, 32, 3, 16)	(16, 16, 3, 32)	(8, 8, 3, 64)	(4, 4,, 3, 64)	(2, 2, 3, 128)	1536×1	$l \times 1$

Encoder network for 3D datasets for Valina autoencoders. l is the desired latent dimension. The input image size is 512×512. For the size of filters, the first two numbers are the width and height of the filter and the third one is for the filter number. The output size has the same format, that is, the numbers are width, height and number of channels.

Using the output of encoder network as input, the decoder network is defined as:

Block	Input	Fully Connected	Reshape	DB3D	DB3D	DB3D	DB3D	DB3D	DB3D	DB3D	Conv3d
Filter size	-	2048	-	(3, 3, 3, 128)	(3, 3, 3, 64)	(3, 3, 3, 32)	(3, 3, 3, 16)	(3, 3, 3, 8)	(3, 3, 3, 4)	(3, 3, 3, 2)	(3, 3, 3, 1)
(a, b)				(2, 1)	(2, 1)	(2, 1)	(2, 1)	(2, 2)	(2, 2)	(2, 2)	
Output size	$l \times 1$	1536×1	(2, 2, 3, 128)	(4, 4, 3, 128)	(8, 8, 3, 64)	(16, 16, 3, 32)	(32, 32, 6, 16)	(64, 64, 6, 8)	(128, 128, 12, 4)	(256, 256, 24, 2)	(256, 256, 24, 1)

Decoder network for 3D datasets for Valina autoencoders. l is the desired latent dimension.

2.3 Network structure for variational autoencoders

The basic network structures for encoders and decoders for variational autoencoders are mostly the same as those for the corresponding Valina autoencoders, except that there are two fully-connected layers to get the output for mean and log of standard deviations, which is the "reparameterization trick" in variational autoencoders [3]. The input for the decoder is the sampled input with "reparameterization trick".

2.4 Network structure for outline autoencoder

The basic structure is a multiple stacks of basic blocks consisting of fully-connected layer, batch normalization layer and ReLu activation layer. The encoder and decoder have four such blocks respectively. The number of filters in the blocks are 2,000, 1,000, 500, 300, respectively for the encoder and in a reversed older for the decoder. In encoder, the input outlines (spharm descriptor) are reshaped as vectors as input for the blocks. The output of the last bock is used as the input for a fully-connected layer to generate the encoded information with given latent dimension. For the decoder, the encoded information is used as input as the blocks and the output from the last block is the input for a fully-connected layer, whose outputs are reshaped to the same shape as the input outlines.

2.5 Parameter setting in the training

Deep autoencoders and variational autoencoders share almost the same hyperparameter setting. The optimizer for deep autoencoders, variational autoencoders and outline autoencoder is adam optimizer [2]. Xavier initializer is applied [1]. The batch size is 100 for autoencoders in 2.1.2, 2.1.3, 2.1.4, 2.3(2D), 2.4 and 10 for that in 2.2.2, 2.3(3D). The number of epoches is 1,000 for autoencoder in 2.1.2, 800 for autoencoders in 2.1.3 and 2.1.4, 5,00 for autoencoder in 2.2.2 and 2.4. A step learning rate scheduler is used for the training. Basically the learning rate is around 1e-3 to 5e-4 in the beginning, and reduces to half around 100-150 epoches before stopping, and finally reduces to around 1e-5 around 50 epoches before stopping. The details are shown in the corresponding scripts in the package.

References

[1] Xavier Glorot and Yoshua Bengio. Understanding the difficulty of training deep feedforward neural networks. In *Proceedings of the thirteenth international conference on artificial intelligence and statistics*, pages 249–256, 2010.

[2] Diederik P Kingma and Jimmy Ba. Adam: A method for stochastic optimization. *arXiv preprint arXiv:1412.6980*, 2014.

[3] Diederik P Kingma and Max Welling. Auto-encoding variational bayes. *arXiv preprint arXiv:1312.6114*, 2013.

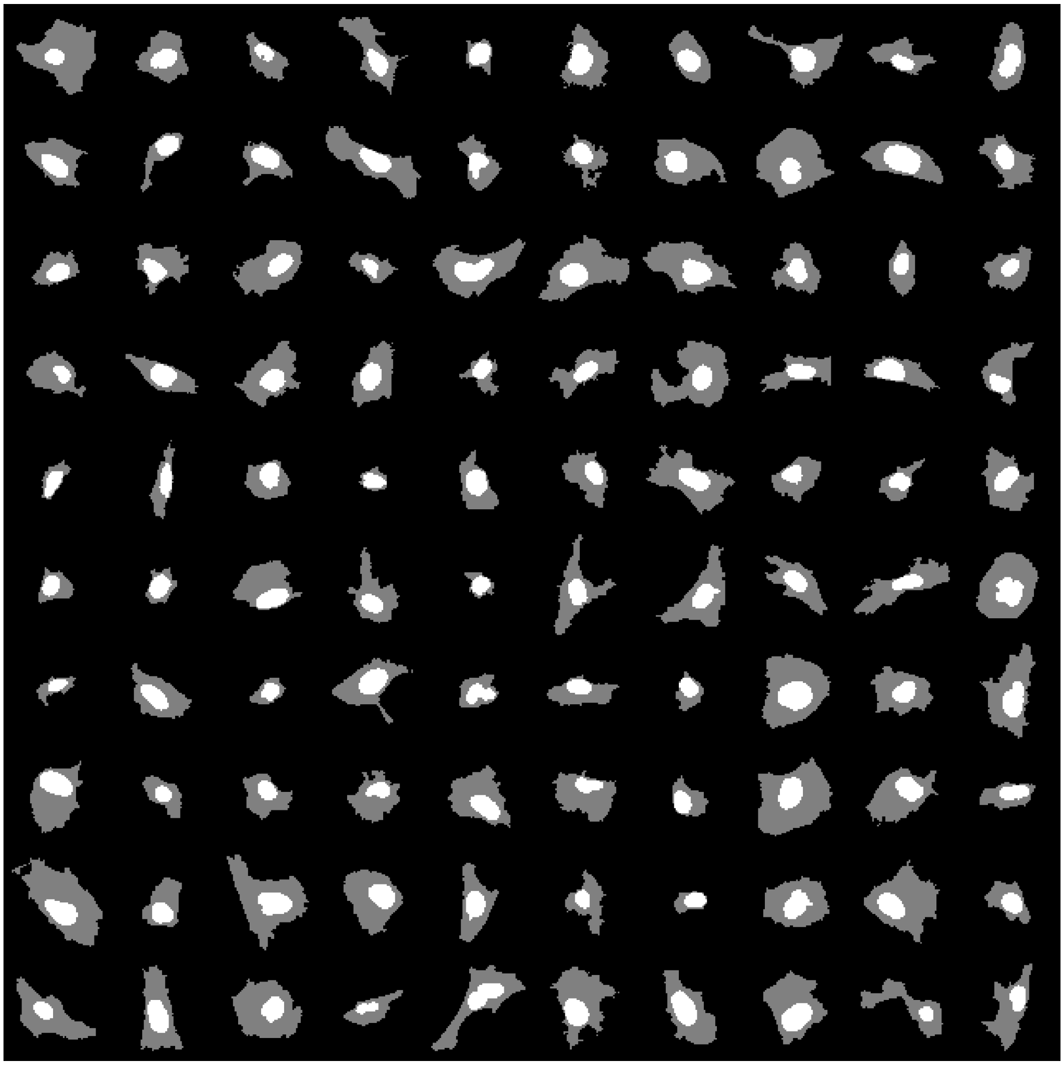
Figure S1: Examples images from the CYTO dataset. 100 randomly chosen cell images are shown.

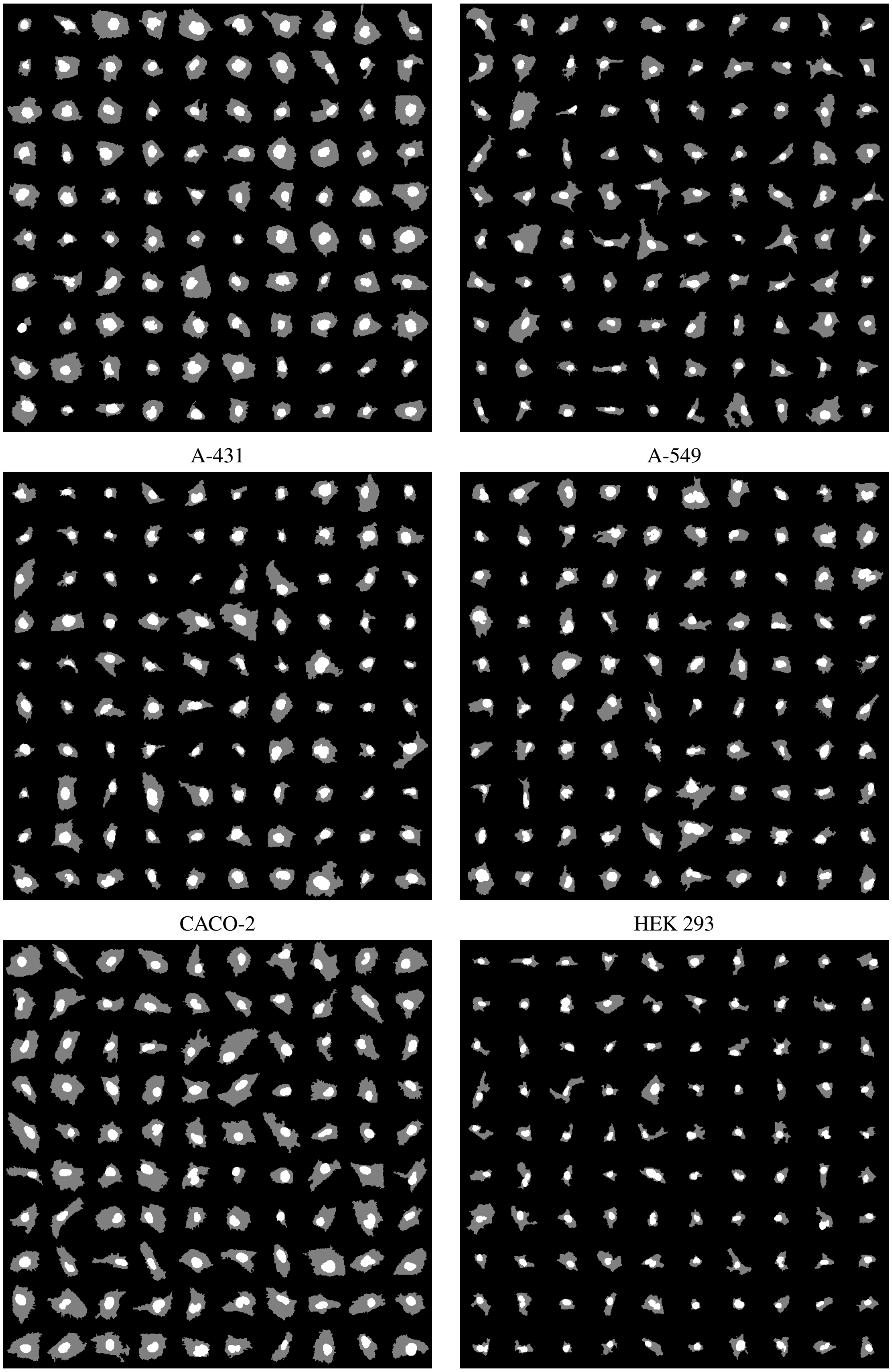

Figure S2: Example images from the HPA cell lines dataset. For each cell line, 100 randomly selected cell images are shown. The name of a cell line is under the corresponding panel.

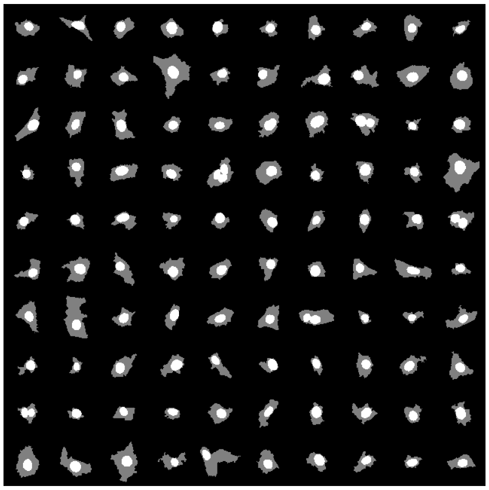

MCF-7

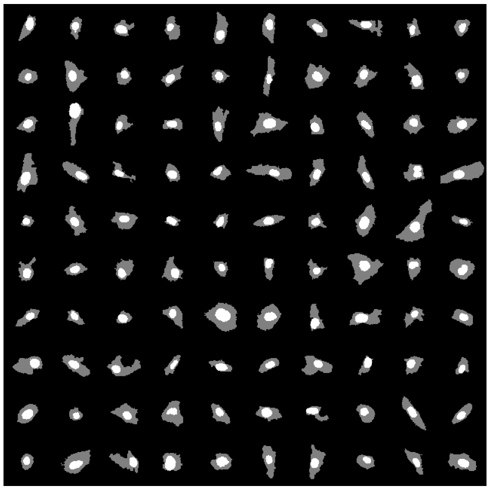

PC-3

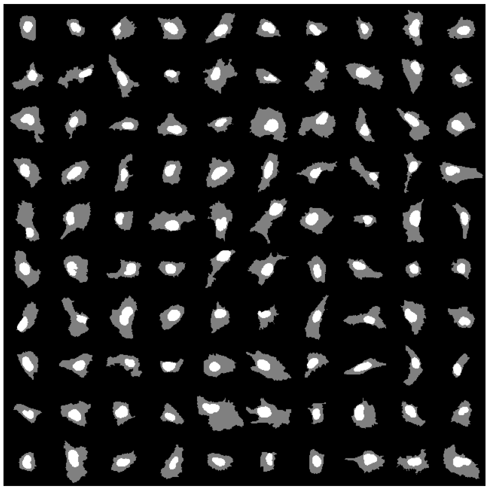

U-251 MG

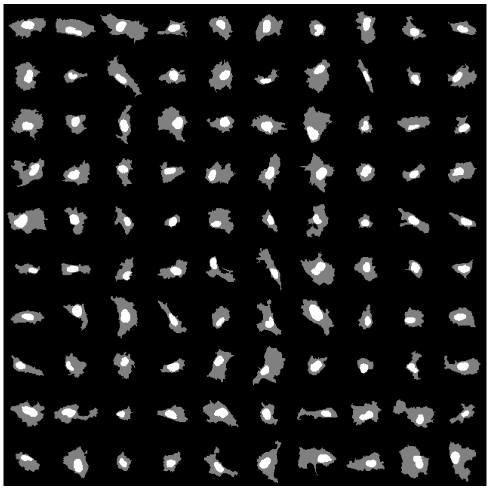

U-2 OS

Figure S2 (Continued): Example images from the HPA cell lines dataset.

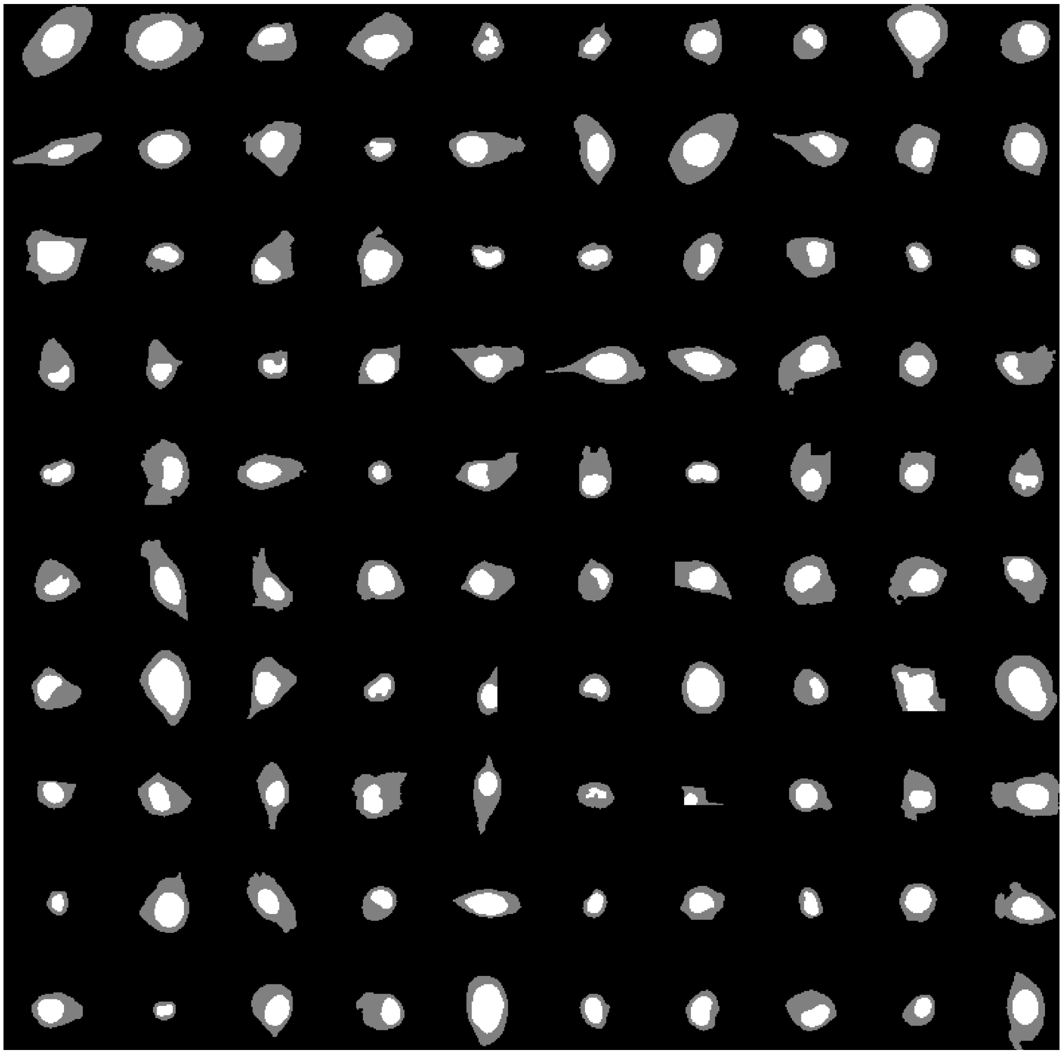

Figure S3: Example images from the H1299 dataset. 100 randomly chosen cell images are shown.

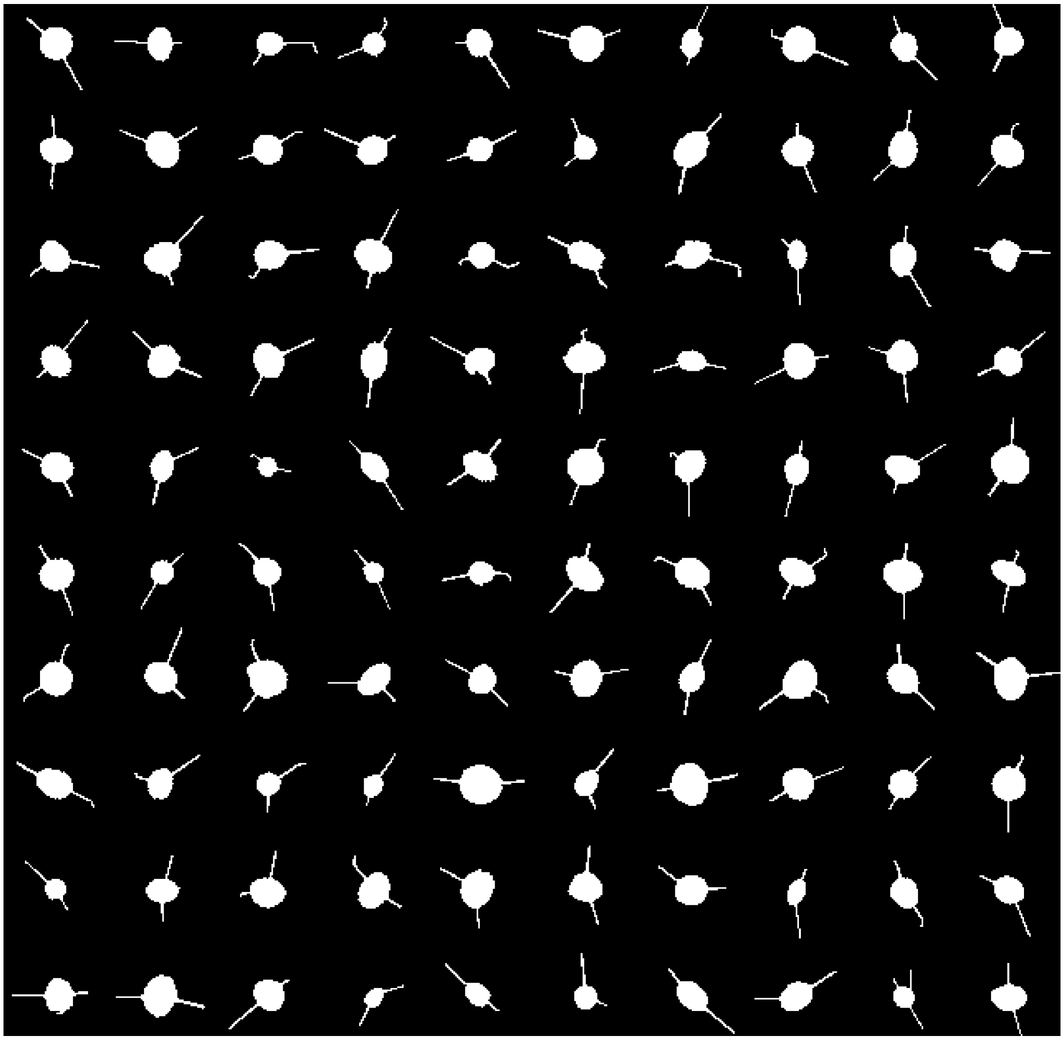

Figure S4: Example images from the SNL dataset. 100 randomly chosen cell images are shown.

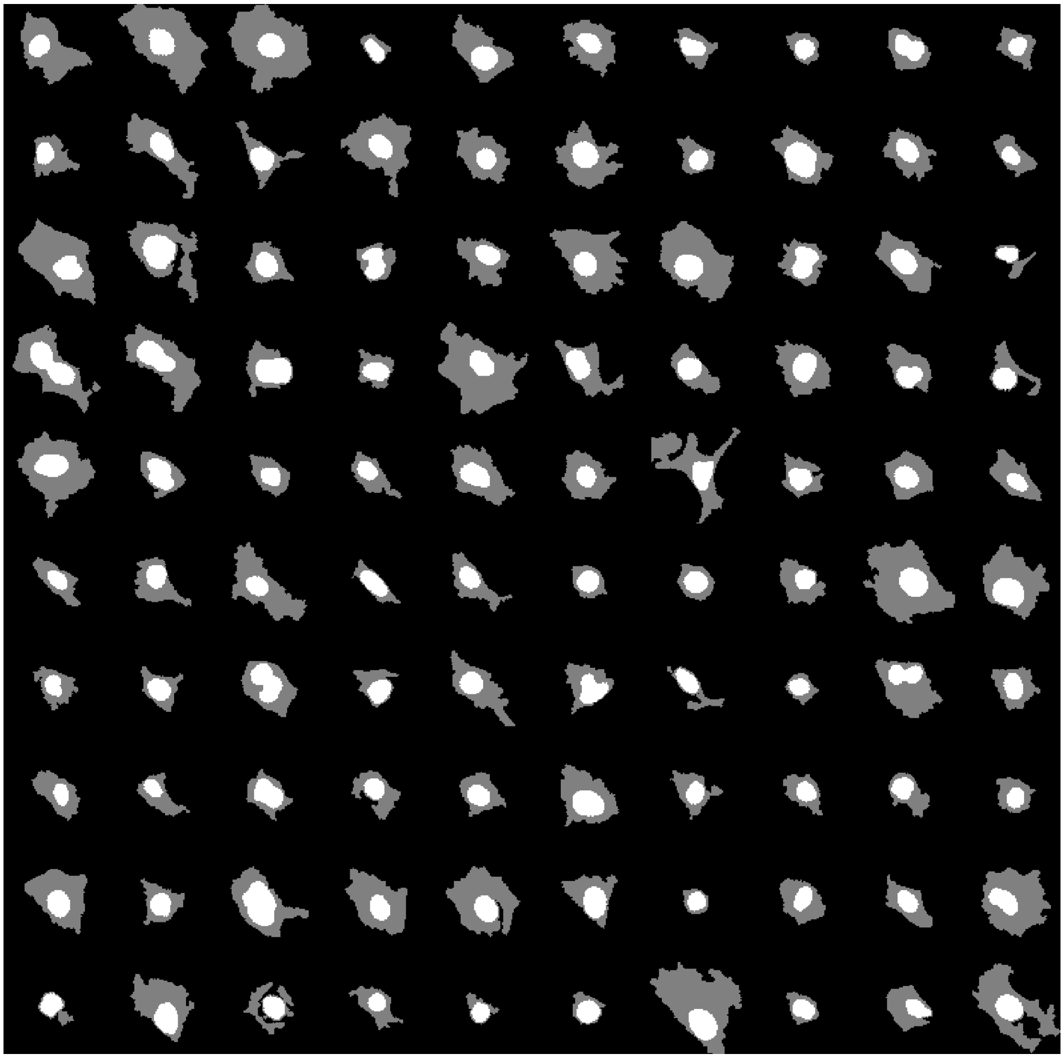

Figure S5: Example images from the MCF7 dataset. 100 randomly chosen cell images are shown.

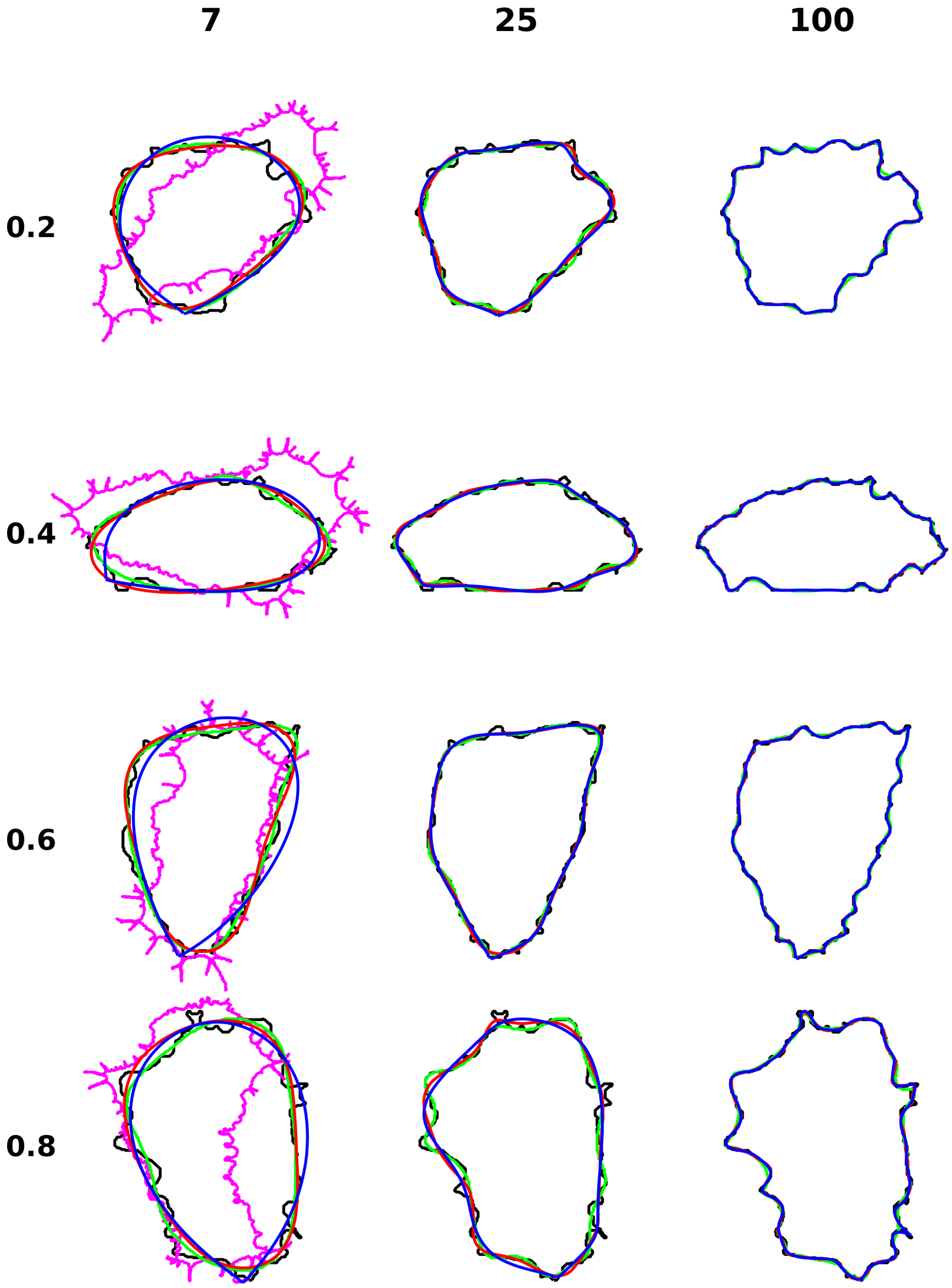

Figure S6: Illustration of representative reconstruction for CYTO dataset. Reconstructions after modeling with different numbers of latent dimensions are shown for cells that have different quantiles of reconstruction errors from PCA model with 100 latent dimensions. Pink color is for diffoemorphic model (only in latent dimension 7), black is the ground truth, blue is for PCA, red is for SCA and green is for autoencoder. The titles are for different latent dimensions and the labels in y-axis are quantiles.

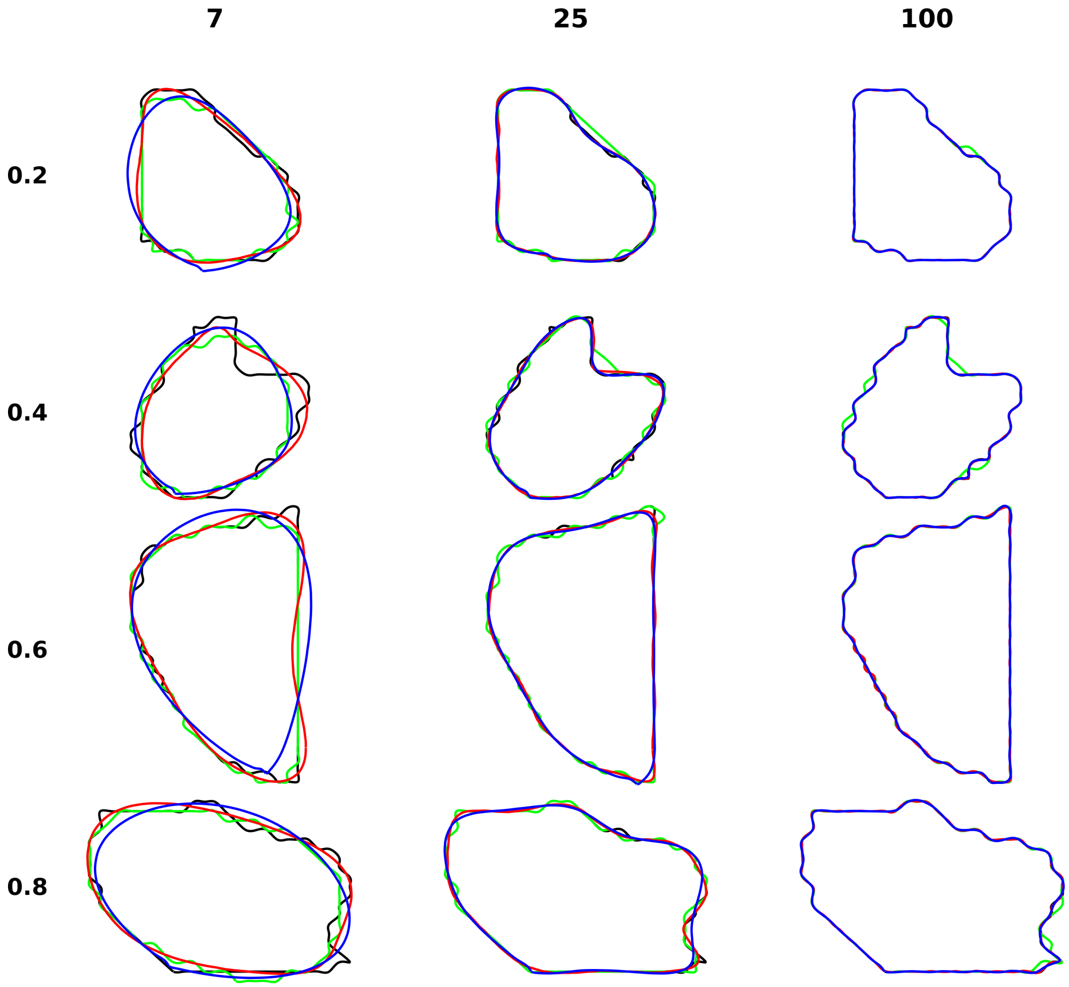

Figure S7: Illustration of representative reconstruction for H1299 dataset. Reconstructions after modeling with different numbers of latent dimensions are shown for cells that have different quantiles of reconstruction errors from PCA model with 100 latent dimensions. Black is the ground truth, blue is for PCA, red is for SCA and green is for autoencoder. The titles are for different latent dimensions and the labels in y-axis are quantiles.

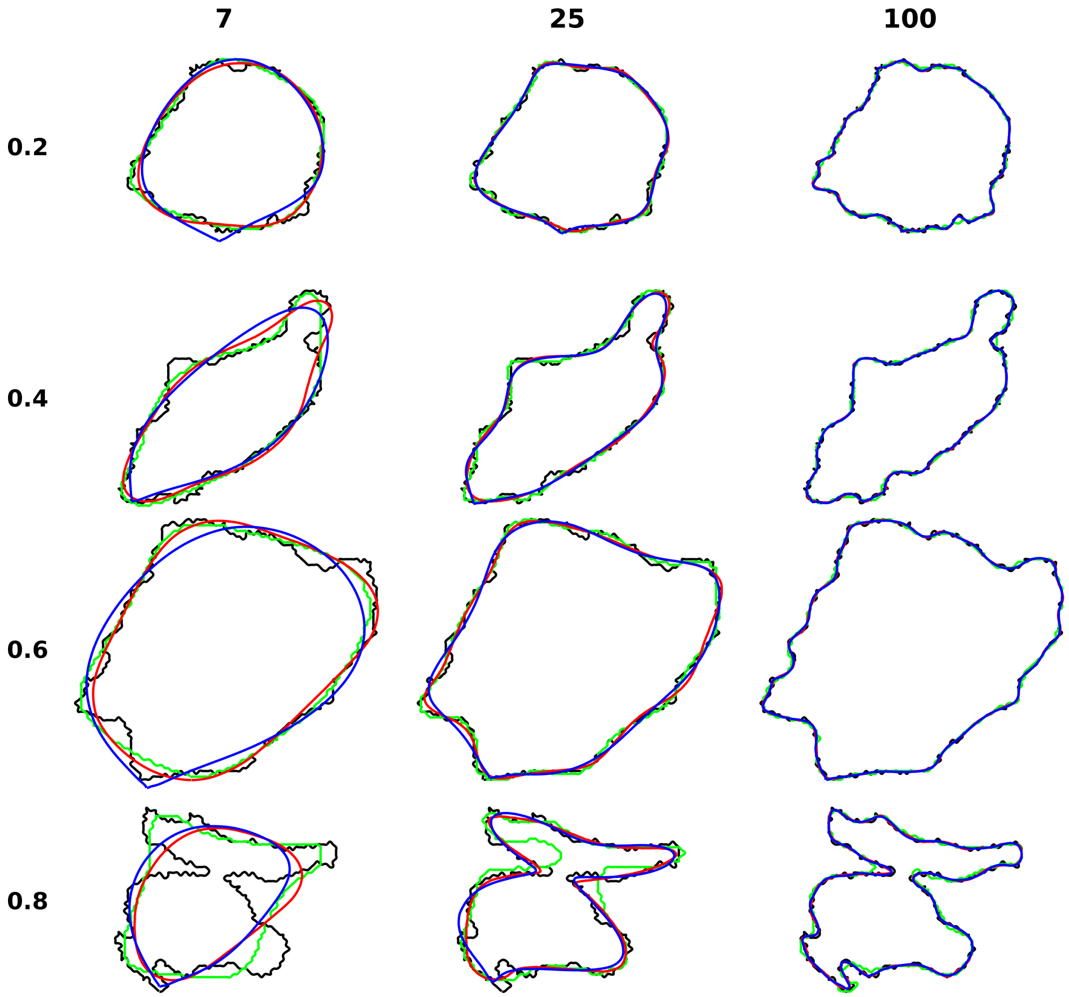

Figure S8: Illustration of representative reconstruction for MCF7 2D dataset. Reconstructions after modeling with different numbers of latent dimensions are shown for cells that have different quantiles of reconstruction errors from PCA model with 100 latent dimensions. Black is the ground truth, blue is for PCA, red is for SCA and green is for autoencoder. The titles are for different latent dimensions and the labels in y-axis are quantiles.

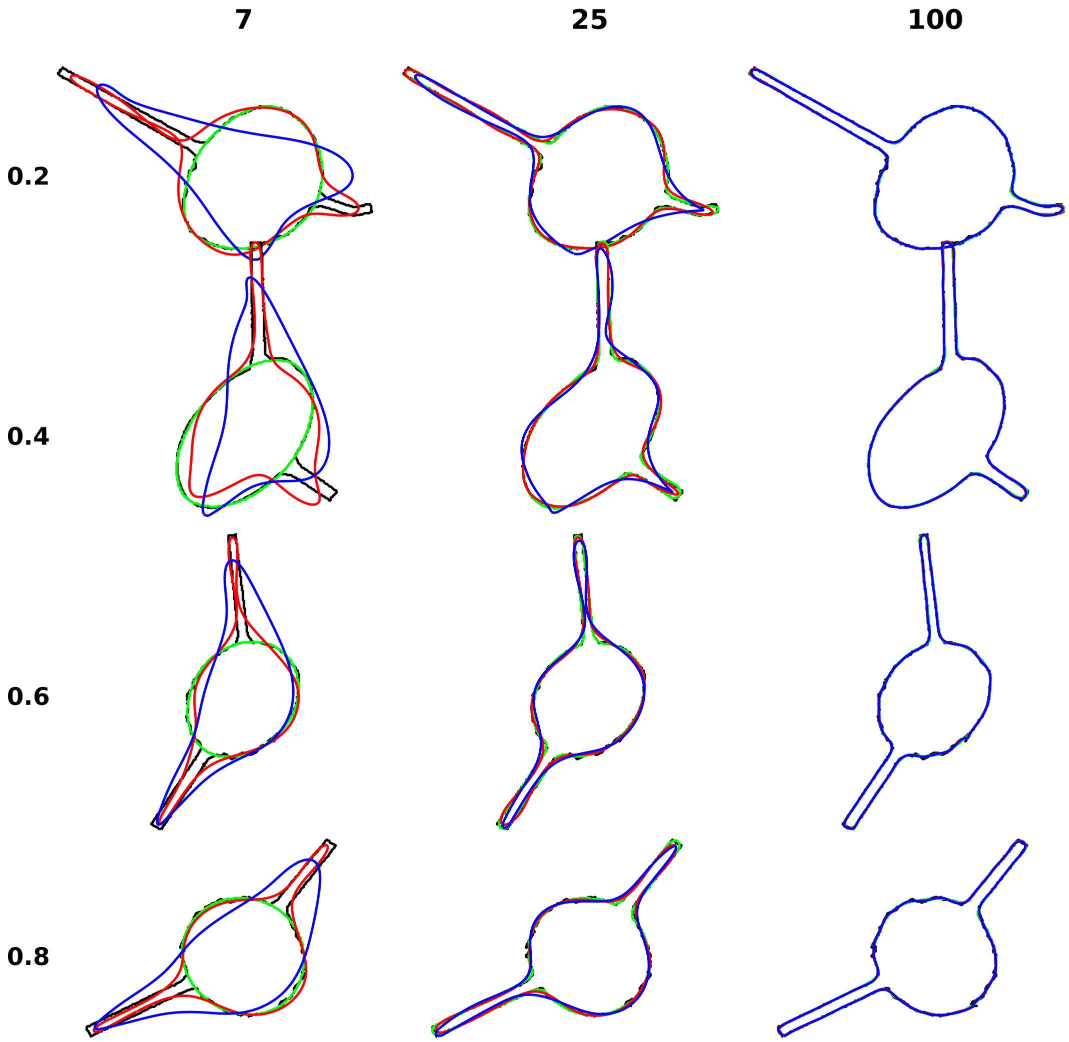

Figure S9: Illustration of representative reconstruction for SNL 2D dataset. Reconstructions after modeling with different numbers of latent dimensions are shown for cells that have different quantiles of reconstruction errors from PCA model with 100 latent dimensions. Black is the ground truth, blue is for PCA, red is for SCA and green is for autoencoder. The titles are for different latent dimensions and the labels in y-axis are quantiles.

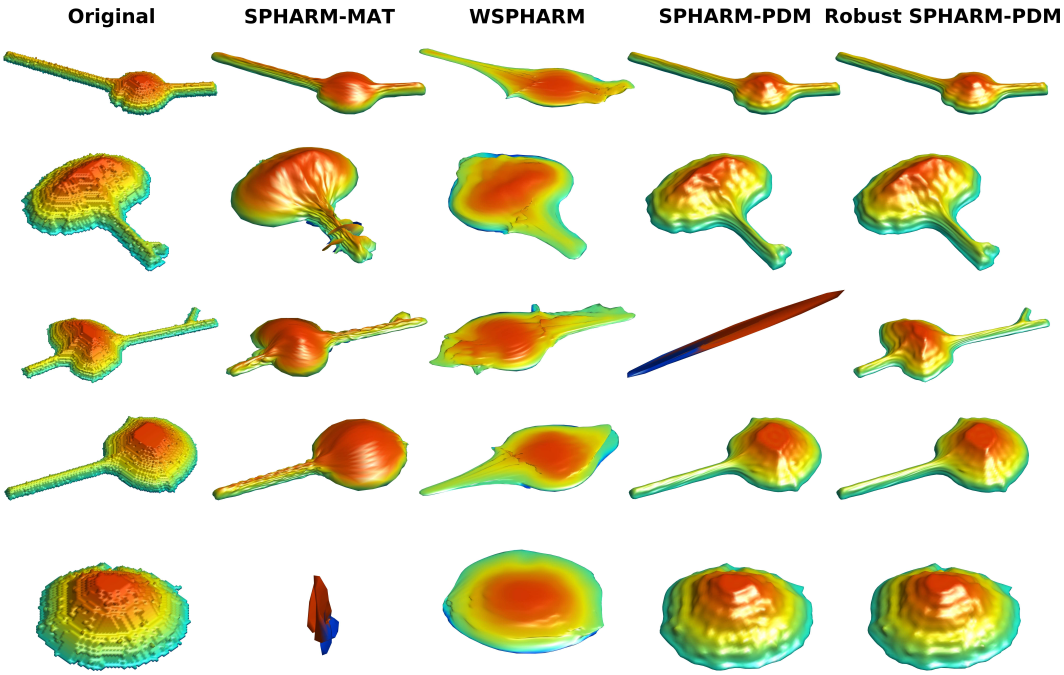

Figure S10: Illustration of SNL 3D parameterization. 5 cells were randomly selected, and the parameterizations for different methods are illustrated with the same cell in the same row. Each face is colored by the order of the z-axis of the its faces.

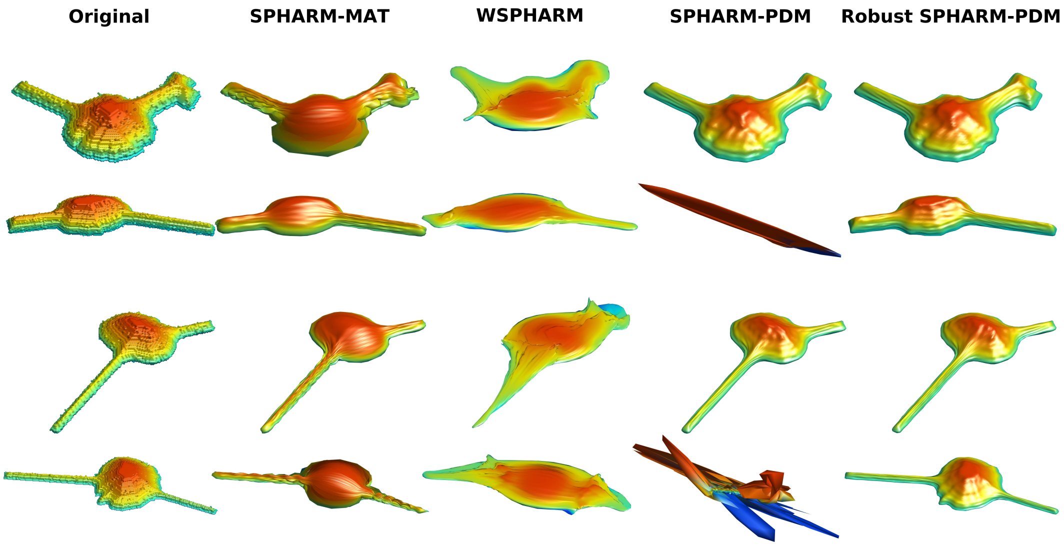

Figure S11: Illustration of SNL NR2 3D parameterization. 4 cells were randomly selected, and the parameterizations for different methods are illustrated with the same cell in the same row. Each face is colored by the order of the z-axis of the its faces.

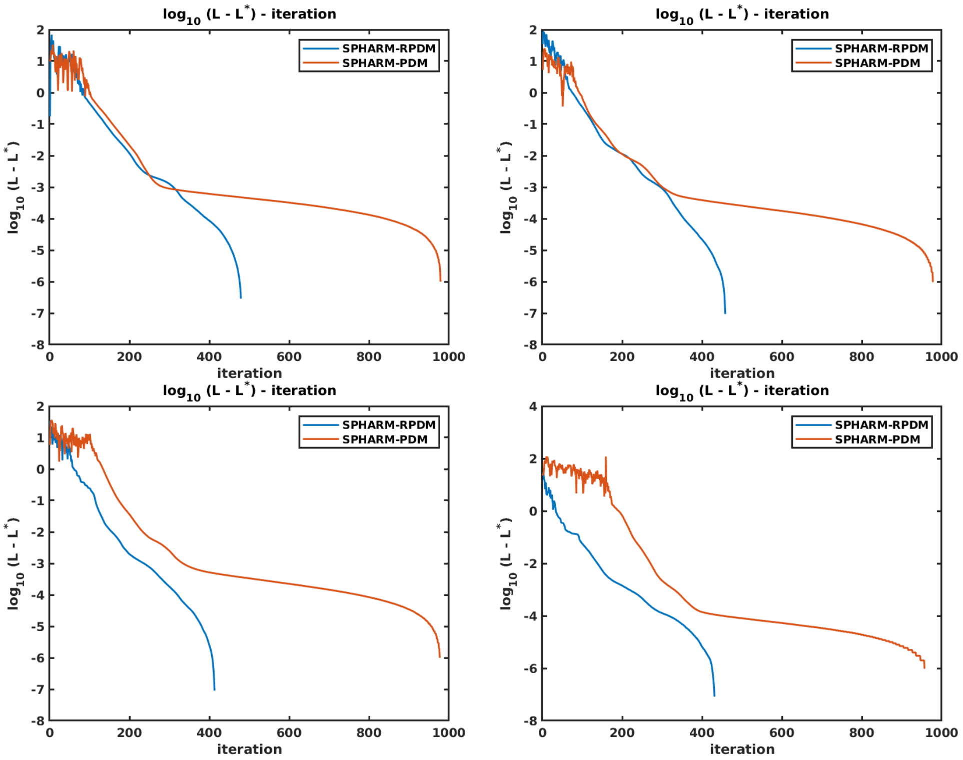

Figure S12: Comparison of convergence process between SPHARM-PDM and SPHARM-RPDM. The parameterization processes for 4 randomly chosen cells are shown. L and L^* are the loss function and the optimal loss (defined as the last iteration when converged), respectively.

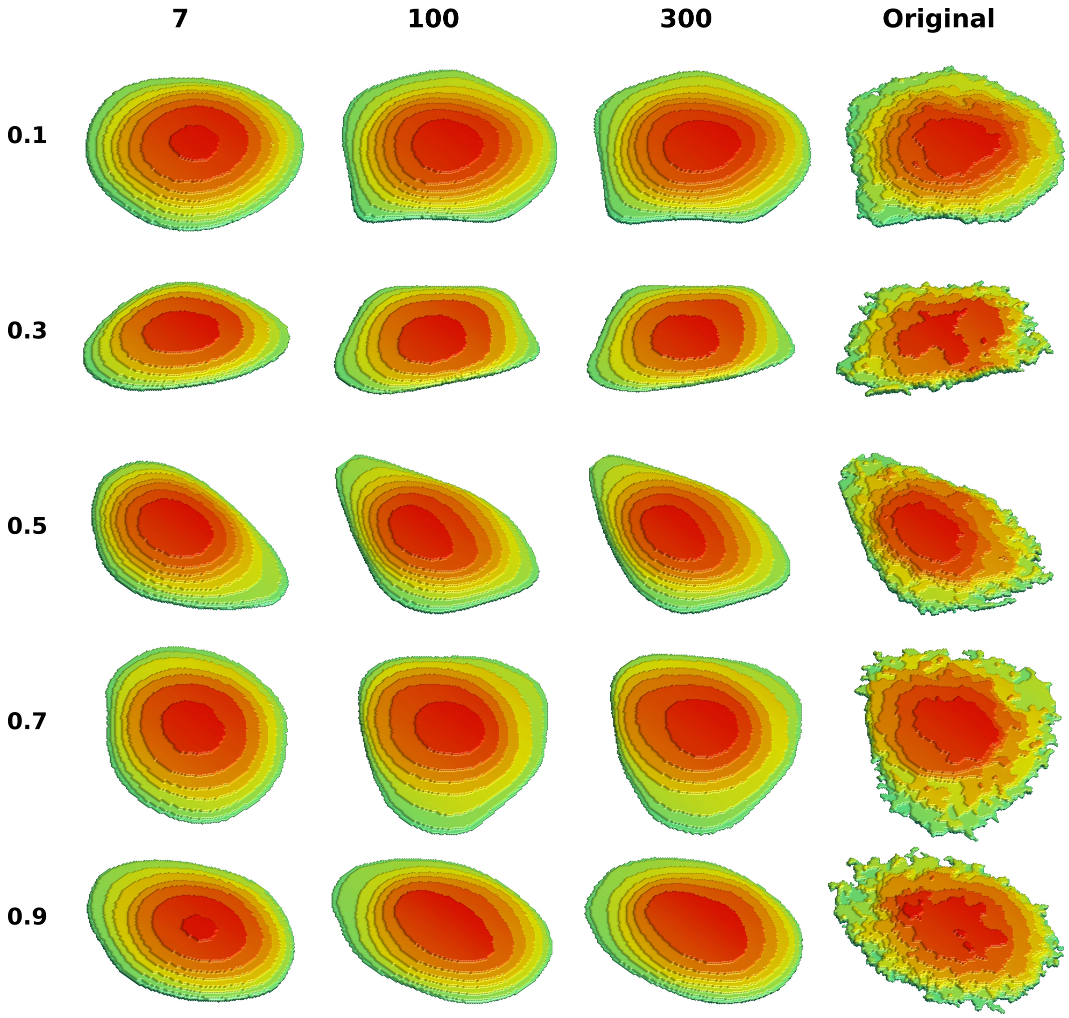

Figure S13: Illustration of Hela 3D reconstruction using a Valina autoencoder. The cells were chosen based on the quantile of reconstruction errors in 300 latent dimensions as listed on the left side. The reconstruction with different numbers of latent dimensions are shown with same cell in the same row, along with the ground truth.

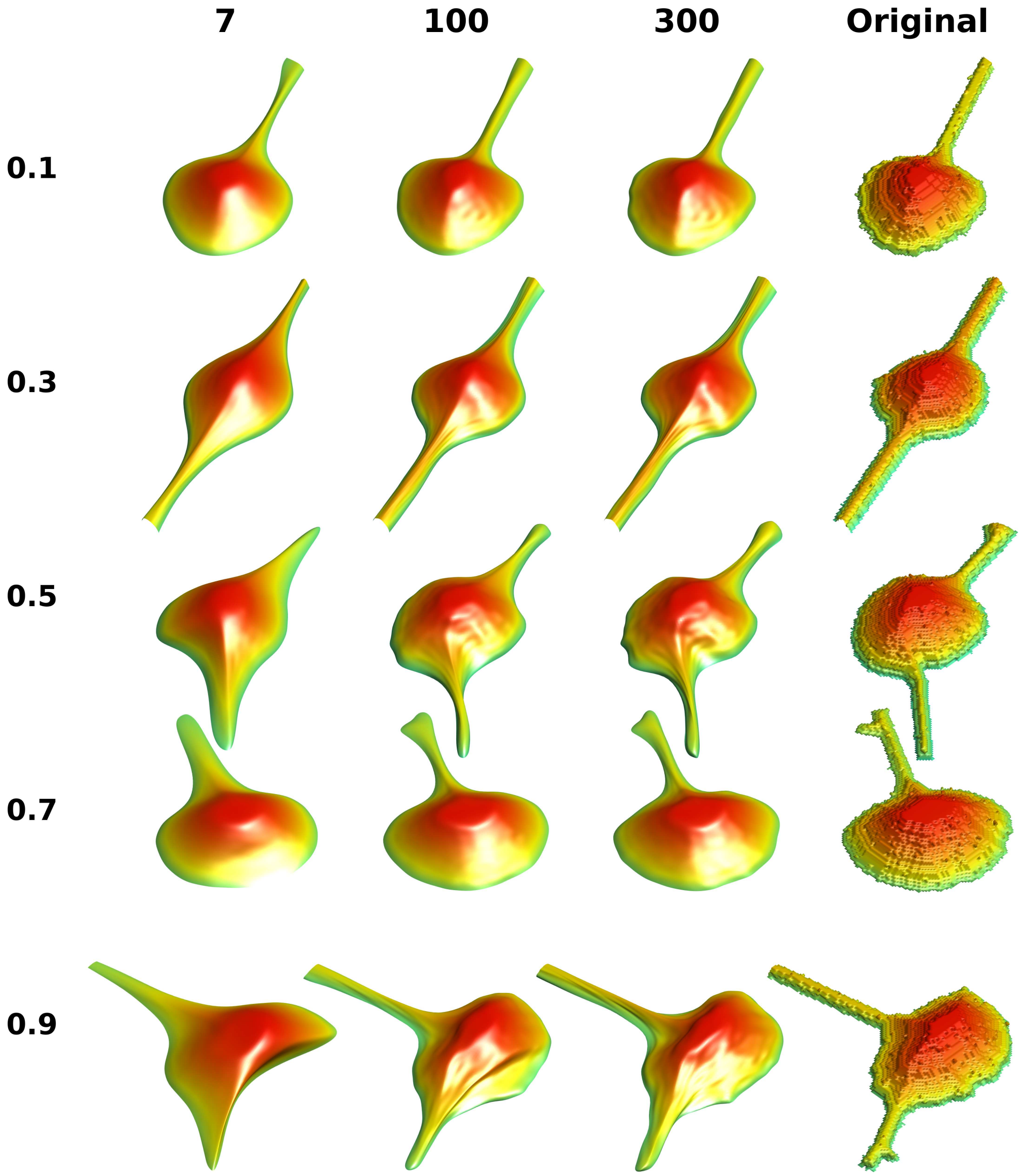

Figure S14: Illustration of SNL 3D reconstruction for SPHARM-SPDM method. The cells were chosen based on the quantile of reconstruction errors in 300 latent dimensions as listed in the left side. The reconstruction of different numbers of latent dimensions are shown with same cell in the same row, along with the ground truth.

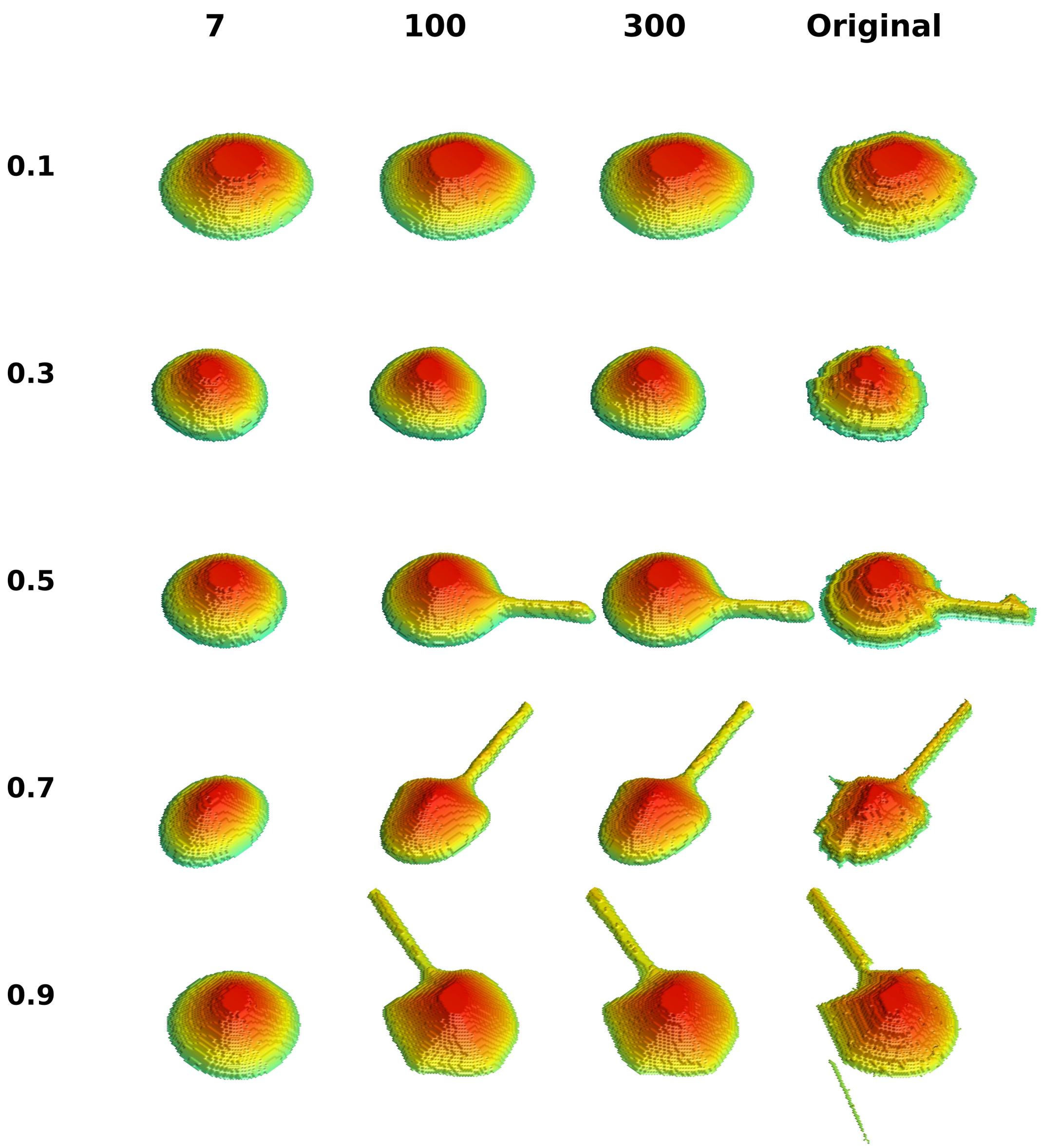

Figure S15: Illustration of SNL 3D reconstruction for Valina autoencoder. The cells were chosen based on the quantile of reconstruction errors in 300 latent dimensions as listed in the left side. The reconstruction of different latent dimensions are shown with same cell in the same row, along with the ground truth.

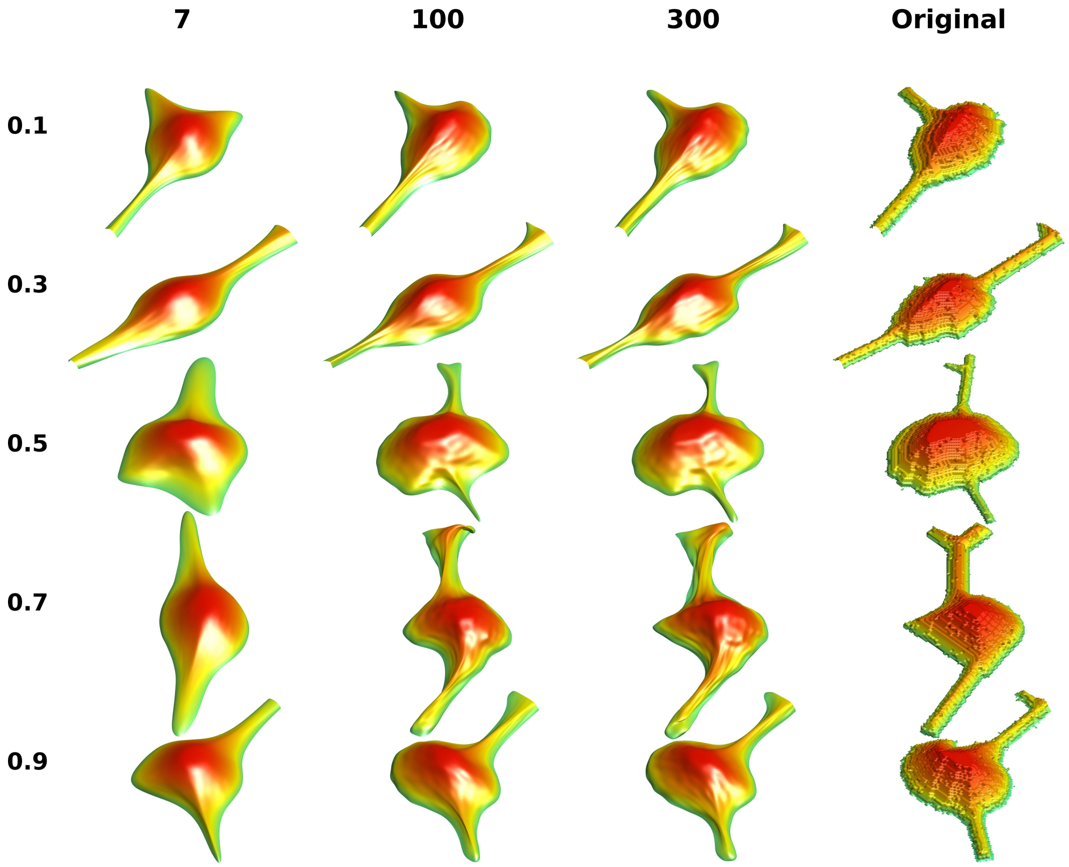

Figure S16: Illustration of SNL NR2 3D reconstruction by the SPHARM-RPDM method. The cells were chosen based on the quantile of reconstruction errors in 300 latent dimensions as listed on the left side. The reconstruction of different latent dimensions are shown with same cell in the same row, along with the ground truth.

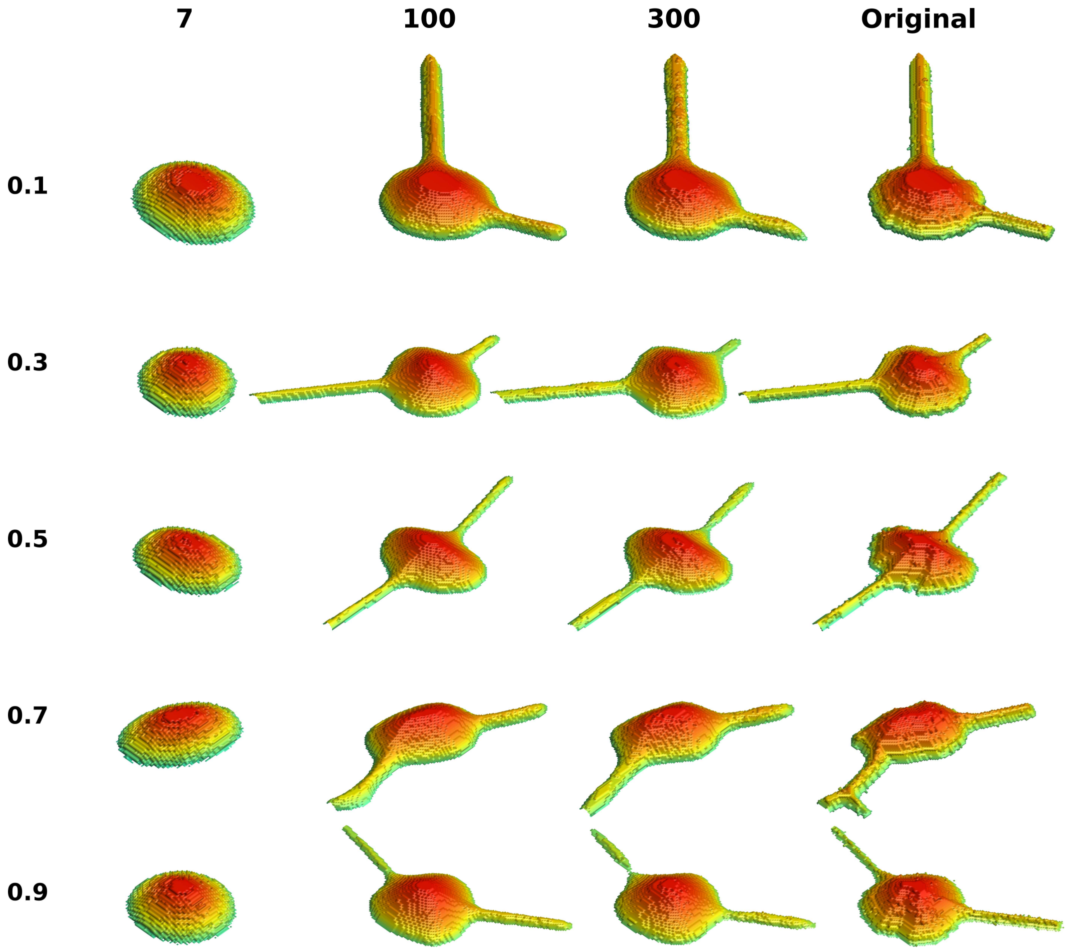

Figure S17: Illustration of SNL NR2 3D reconstruction for Valina autoencoder. The cells were chosen based on the quantile of reconstruction errors in 300 latent dimensions as listed on the left side. The reconstruction of different latent dimensions are shown with same cell in the same row, along with the ground truth.

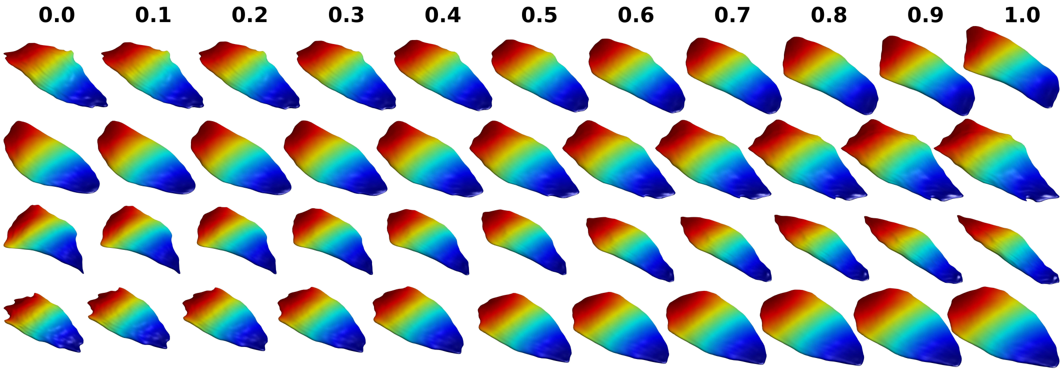

Figure S18: Illustrations of shape evolutions for HeLa dataset. Four pairs of cells were randomly selected. The source, target and intermediate shapes in the linear path between the chosen cells are shown, with the title showing the relative distance to the source.

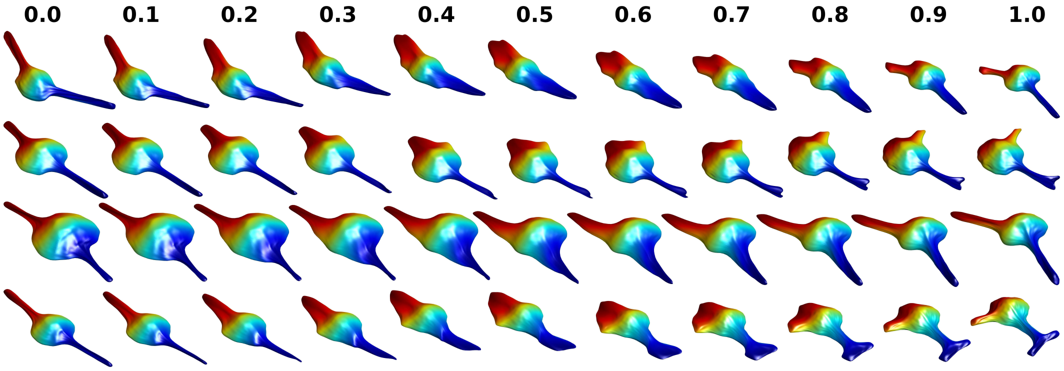

Figure S19: Illustrations of shape evolutions for SNL NR2 3D dataset. Four pairs of cells were randomly selected. The source, target and intermediate shapes in the linear path between them are shown, with the title showing the relative distance to the source.

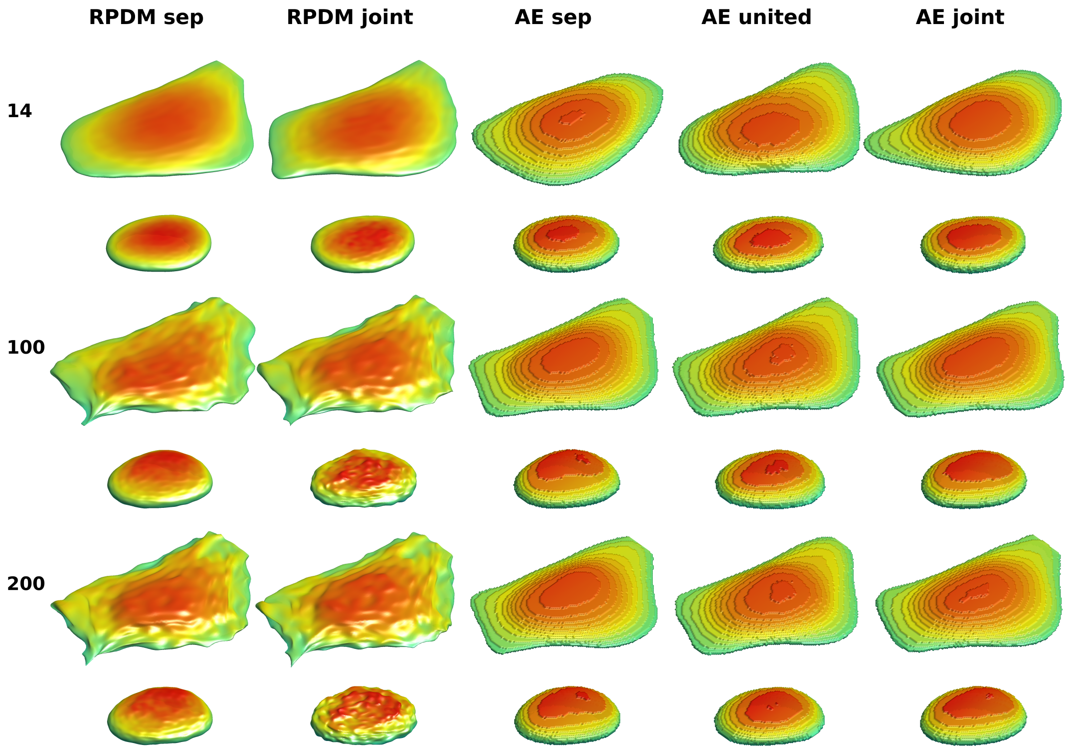

Figure S20: Illustration of joint modeling of 3D HeLa cell and nuclear shapes using different methods. We chose a cell in the 1.0 quantile of the joint reconstruction errors (worst reconstruction) for SPHARM-RPDM joint models with 200 latent dimensions. This cell is shown across different methods and different numbers of latent dimensions, which are indicated in the title and y-axis, respectively.

		PCA	SCA	Diffeo-morphic	AE	SRAE	VAE	OAE
7	CYTO	0.174	0.249	0.744	0.157	0.684	0.159	0.170
	HPA CL	0.164	0.240	0.436	0.152	0.309	0.157	0.161
	H1299	0.097	0.118	–	0.055	0.092	0.184	0.143
	MCF7	0.180	0.535	–	0.136	0.447	0.139	0.176
	SNL	0.204	0.237	–	0.165	0.714	0.166	0.094
100	CYTO	0.025	0.026	–	0.038	0.142	0.048	0.112
	HPA CL	0.024	0.025	–	0.034	0.068	0.046	0.103
	H1299	0.089	0.087	–	0.020	0.108	0.336	0.116
	MCF7	0.042	0.030	–	0.037	0.082	0.067	0.118
	SNL	0.029	0.043	–	0.025	0.075	0.049	0.068

Table S1: Pixel level reconstruction errors for 2D shapes for the four datasets. Due to its extremely long computing time and inability to sample shapes in high dimensional space, we only show the results for 7 dimensions for CYTO and HPA cell lines for diffeomorphic model. The computing times shown here are CPU time for PCA and diffeomorphic models and GPU time for deep autoencoders.

Dim	Datasets	PCA				SCA				AE						SRAE			
		sep		joint		sep		joint		sep		united		joint		sep		joint	
		cell	nuc	cell	nuc	cell	nuc	cell	nuc	cell	nuc	cell	nuc	cell	nuc	cell	nuc	cell	nuc
14	CYTO	26.2	6.02	22.1	11.2	31.7	9.40	31.2	10.1	34.5	5.86	27.1	8.10	31.6	8.30	66.5	5.86	52.0	59.6
	HPA CL	24.2	5.70	20.4	10.4	29.6	8.81	29.1	9.46	30.2	5.61	24.2	7.71	27.3	7.87	55.9	5.61	38.6	26.8
	H1299	1.22	1.19	1.12	1.58	1.56	1.83	1.56	1.80	1.62	9.24	1.35	1.41	1.46	1.41	1.89	9.24	1.74	2.06
	MCF7	8.63	1.86	7.24	3.18	9.48	2.56	9.43	2.66	8.93	1.72	7.44	2.31	8.59	1.86	23.2	1.72	9.09	3.77
200	CYTO	4.20	0.655	2.49	1.56	4.41	0.678	2.65	1.60	10.5	1.80	8.09	2.85	9.86	2.05	34.0	1.80	16.9	11.4
	HPA CL	3.76	0.646	2.24	1.49	3.95	0.668	2.37	1.53	8.84	1.76	6.98	2.77	8.15	2.01	15.9	1.76	14.5	6.82
	H1299	0.219	0.118	0.170	0.218	0.288	0.110	0.190	0.240	0.626	14.9	0.950	1.08	3.58	4.60	1.78	14.9	2.13	2.30
	MCF7	1.38	0.460	0.962	0.894	1.41	0.627	0.989	0.888	2.70	1.14	2.68	1.33	2.80	1.18	5.44	1.14	6.26	2.94

Table S2: Reconstruction errors of cell and nuclear errors in joint modeling. The table shows two latent dimensions 14 and 200 for the four 2D datasets for four representative methods. 'sep', 'joint' and 'united' means separate models, joint models and united models for corresponding methods as described in Methods.

Dim	SPHARM-RPDM				AE						SRAE			
	sep		joint		sep		united		joint		sep		joint	
	cell	nuc	cell	nuc	cell	nuc	cell	nuc	cell	nuc	cell	nuc	cell	nuc
14	8.38	3.17	8.03	3.40	16.2	3.20	107	4.49	13.5	3.42	16.9	3.20	26.1	NaN
200	4.89	2.00	4.64	2.20	7.93	2.32	7.85	3.26	8.88	3.16	16.9	2.32	20.4	NaN

Table S3: Reconstruction errors of cell and nuclear errors in joint modeling. The table shows two latent dimensions 14 and 200 for the four 2D datasets for four representative methods. ‘sep’, ‘joint’ and ‘united’ means separate models, joint models and united models for corresponding methods as described in Methods.
